# Comparative analysis of the complete chloroplast genome sequences of six species of *Pulsatilla* Miller, Ranunculaceae

**DOI:** 10.1186/s13020-019-0274-5

**Published:** 2019-11-28

**Authors:** Tingting Zhang, Yanping Xing, Liang Xu, Guihua Bao, Zhilai Zhan, Yanyun Yang, Jiahao Wang, Shengnan Li, Dachuan Zhang, Tingguo Kang

**Affiliations:** 1 School of Pharmacy, Liaoning University of Traditional Chinese Medicine, Dalian, China; 2 Liaoning Quality Monitoring and Technology Service Center for Chinese Materia Medica Raw Materials, Dalian, China; 3 School of Mongol Medicine, Inner Mongolia University for Nationalities, Tongliao, China; 4 Traditional Chinese Medicine Resource Center, Chinese Academy of Traditional Chinese Medicine, Beijing, China

**Keywords:** *Pulsatilla chinensis*, *Pulsatilla* Miller, Chloroplast genome, Phylogeny

## Abstract

**Background:**

Baitouweng is a traditional Chinese medicine with a long history of different applications. Although referred to as a single medicine, Baitouweng is actually comprised of many closely related species. It is therefore critically important to identify the different species that are utilized in these medicinal applications. Knowledge about their phylogenetic relationships can be derived from their chloroplast genomes and may provide additional insights into development of molecular markers.

**Methods:**

Genomic DNA was extracted from six species of *Pulsatilla* and then sequenced on an Illumina HiSeq 4000. Sequences were assembled into contigs by SOAPdenovo 2.04, aligned to the reference genome using BLAST, and then manually corrected. Genome annotation was performed by the online DOGMA tool. General characteristics of the cp genomes of the six species were analyzed and compared with closely related species. Additionally, phylogenetic trees were constructed, based on single nucleotide polymorphisms (SNPs) and 51 shared protein-coding gene sequences in the cp genome among all 31 species via maximum likelihood.

**Results:**

The size of cp genomes of *P*. *chinensis* (Bge.) Regel, *P. chinensis* (Bge.) Regel var. *kissii* (Mandl) S. H. Li et Y. H. Huang, *P. cernua* (Thunb.) Bercht. et Opiz f. *plumbea* J. X. Ji et Y. T. zhao, *P. dahurica* (Fisch.) Spreng, *P. turczaninovii* Kryl. et Serg, and *P. cernua* (Thunb.) Bercht. et Opiz. were 163,851 bp, 163,756 bp, 162,481 bp, 162,450 bp, 162,795 bp, and 162,924 bp, respectively. Each species included two inverted repeat regions, a small single-copy region, and a large single-copy region. A total of 134 genes were annotated, including 90 protein-coding genes, 36 tRNAs, and eight rRNAs across all species. In simple sequence repeat analysis, only *P. dahurica* was found to contain hexanucleotide repeats. A total of 26, 39, 32, 37, 32 and 43 large repeat sequences were identified in the genic regions of the six *Pulsatilla* species. Nucleotide diversity analysis revealed that the *rpl36* gene and *ccsA*-*ndhD* region have the highest Pi value. In addition, two phylogenetic trees of the cp genomes were constructed, which laced all *Pulsatilla* species into one branch within Ranunculaceae.

**Conclusions:**

We identified and analyzed the cp genome features of six species of *P.* Miller, with implications for species identification and phylogenetic analysis.

## Background

Baitouweng is the dry root of *Pulsatilla chinensis*, Ranunculaceae. It is a traditional Chinese medicine that has been used to alleviate fever and treat dysentery [1]. A total of 43 species have been identified in Europe and Asia, with 11 found in China [2]. Triterpenoid saponins are thought to be one of the main active components in Baitouweng [3]. It is included in the *Chinese Pharmacopoeia* as a genuine medicinal material, but not all species are included. Our previous investigation into the market of Chinese medicine found that there are many counterfeits, partially due to mistaking closely related species. This seriously affects the quality of medicinal materials and clinical efficacy. Previously, our team has studied DNA barcodes of *Pulsatilla* [4]. However, there are limited studies on the phylogenetic position and species diversity of these species, which could be improved by focusing on their chloroplast (cp) genomes.

The chloroplast is an important organelle in plants, which provides energy through photosynthesis and plays an important role in carbon uptake. Additionally, it contains its own genome, which takes the form of a cyclic double-stranded DNA molecule, with a maternal inheritance pattern [5–8]. Typical cp genome structure consists of four stable parts—two inverted repeats (IRs), a large single copy (LSC) region, and a small single copy (SSC) region [7]. In general, the cp genome contains an average of about 120 kb of unique sequences. In addition to the rRNA and tRNA genes, the number of protein-coding genes in the cp genome is about 100 [9]. In recent years, molecular identification has been widely used to discern true Chinese medicines from their counterfeits [10, 11]. With the rapid development of next generation sequencing, the acquisition of genomes is faster and cheaper than traditional Sanger sequencing [12]. Compared with nuclear genome DNA, cp genome DNA has a low molecular weight, multiple copies, and a simple structure, which are conducive to cp genome analysis [13]. Simple sequence repetition (SSR) has high mutation rate and multiple copies, and SSR markers have been widely used in genetic diversity and evolutionary research [14, 15].

In this study, the cp genomes of *P. chinensis*, *P. chinensis* var. *kissii*, *P. cernua* f. *plumbea*, *P. dahurica*, *P. turczaninovii*, and *P. cernua* were sequenced to analyze their structures and explore differences at molecular level. Meanwhile, compared with the cp genomic characteristics of *Aconitum carmichaelii* (NC_030761.1) and *Coptis chinensis* (NC_036485.1), whether there was a characteristic variation. We also analyzed SSRs, large sequence repeats, IR boundaries, and nucleotide diversity in an attempt to identify differences. Phylogenetic analysis was carried out to determine the evolutionary relationship and phylogenetic positions of six *Pulsatilla* species.

## Methods

### DNA extraction and sequencing

Fresh leaves of six species of *Pulsatilla* were collected from Liaoning Provincial Preservation Nursery of Key Species of Chinese Medicinal Plants in Dalian Campus of Liaoning University of Traditional Chinese Medicine (N 39°06′, E 121°87′, Dalian, Liaoning Province, China). *P. chinensis* was introduced in Dalian, Liaoning Province. *P. chinensis* var*. kissii* was introduced in Anshan, Liaoning Province. *P. cernua* f. *plumbea* was introduced in Jiaohe, Jilin Province. *P. dahurica* was introduced in Yichun, Heilongjiang Province. *P. turczaninovii* was introduced in Tongliao, Inner Mongolia Autonomous Region. *P. cernua* was introduced in Dandong, Liaoning Province. All the species were introduced by Xu Liang. Professor Kang Tingguo at Liaoning University of Traditional Chinese Medicine, identified the certificate specimens (*P. chinensis* 10162180425513LY, *P. chinensis* var*. kissii* 10162180429514LY, *P. cernua* f. *plumbea* 10162180503515LY, *P. dahurica* 10162180503516LY, *P. turczaninovii* 10162180504517LY, *P. cernua* 10162180504518LY) and deposited them in the Herbarium of Liaoning University of Traditional Chinese Medicine. Approximately 5 g of fresh leaves was harvested for cp DNA isolation using a modified cetyl trimethylammonium bromide method [16]. After DNA isolation, 1 μg of purified DNA was fragmented and used to construct short-insert libraries (insert size 430 bp) according to the manufacturer’s instructions (Illumina), then sequenced on the Illumina Hiseq 4000 [17].

### Genome assembly and annotation

Prior to assembly, raw reads were filtered to remove reads with adaptor contamination, low quality (Q < 20), or a high percentage of uncalled bases (> 10%). The cp genome was reconstructed using a combination of de novo and reference-guided assemblies [18]. First, the filtered reads were assembled into contigs using SOAP denovo 2.04 [19]. Contigs were then aligned to the reference genome using BLAST and aligned contigs (≥ 80% similarity and query coverage) were ordered according to the reference genome. Finally, the clean reads were mapped to the assembled draft cp genome for base correction, and most gaps were filled through local assembly. The cp genes were annotated using an online DOGMA tool [20] with default parameters to predict protein-coding genes, transfer RNA (tRNA) genes, and ribosome RNA (rRNA) genes. A whole cp genome BLAST [21] search was performed against five databases, with cutoffs of < 1e^−5^ E-value and minimum alignment length percentage of > 40%. Searched databases included KEGG (Kyoto Encyclopedia of Genes and Genomes) [22–24], COG (Clusters of Orthologous Groups) [25, 26], NR (Non-Redundant Protein Database databases), Swiss-Prot [27], and GO (Gene Ontology) [28]. The sequencing data and gene annotations were then submitted to GenBank and assigned accession numbers (*P. chinensis*: MK860682, *P. chinensis* var*. kissii*: MK860683, *P. cernua* f. *plumbea*: MK860684, *P. dahurica*: MK860685, *P. turczaninovii*: MK860686, *P. cernua*: MK860687).

The cp genomes were then mapped using Organellar Genome Draw (OGDRAW) (Max Planck Institute of Molecular Plant Physiology, Am Mühlenberg, Potsdam, Germany) (http://ogdraw.mpimp-golm.mpg.de/index.Shtml) [29].

### Comparative analysis of the cp genomes

The SSR software MicroSAtellite (MISA) (http://pgrc.ipk-gatersleben.de/misa/) was used to identify SSR sequences and tandem repeats of 1–6 nucleotides were considered microsatellites. The minimum numbers of repeats were set to 10, 6, 5, 5, 5, and 5 for mono-, di-, tri-, tetra-, penta-, and hexa-nucleotides, respectively. The maximum number of bases interrupting two SSRs in a compound microsatellite was set to 100. The data were then compared with *A*. *carmichaelii* (NC_030761.1) and *C*. *chinensis* (NC_036485.1), with an emphasis on perfect repeat sequences [30]. Web-based REPuter (http://bibiserv.techfak.uni-bielefeld.de/reputer/) was used to analyze the long repeat sequences, which included forward, reverse, complement, and palindromic repeats with minimum sequence length of 30 bp and edit distances of 3 bp [31]. DnaSP v5.10 was utilized to determine the average number of nucleotide differences between the six genomes [32].

### Phylogenetic analysis

In order to analyze the relationship between the phylogenetic position of *Pulsatilla* and other genera in Ranunculaceae, phylogenetic trees were constructed by aligning cp genome sequences from 31 species, 25 of which were obtained from GenBank. Among the 31 species, there were two outgroups: *Arabidopsis thaliana* (NC_003076.8) and *Panax ginseng* (NC_006290.1). Phylogenetic analysis included 29 species in Ranunculaceae, one species of Cruciferae, and one species of Araliaceae. Single nucleotide polymorphisms (SNPs) and 51 shared protein-coding genes of the cp genome for all 31 species were analyzed. The PhyML V3.0 software was used to construct a phylogenetic tree by maximum likelihood method (ML), and a model GTR+I+G was selected for ML analyses with 1000 bootstrap replicates to calculate bootstrap values [33].

## Results and discussion

### General characteristics

The cp genome sizes of the six species were 163,851, 163,756, 162,481, 162,450, 162,795 and 162,924 bp for *P. chinensis*, *P. chinensis* var. *kissii*, *P. cernua* f. *plumbea*, *P. dahurica*, *P. turczaninovii*, and *P. cernua*, respectively. The similar sizes of these genomes indicate that they were highly conserved during the course of evolution. The overall GC content of the *P. chinensis*, *P. chinensis* var. *kissii*, *P. cernua* f. *plumbea*, *P. dahurica*, *P. turczaninovii*, and *P*. cernua cp genomes was 37.14%, 37.16%, 37.42%, 37.42%, 37.40%, and 37.35%, respectively. The minor differences in cp genome size is mainly caused by changes in the LSC region. Most variations occur in the non-coding regions, which has been reported in other systems previously [34]. It is reflected in the species of *Cerasus humilis* [35], *Talinum paniculatum* [36] and *Heimia myrtifolia* [37]. The largest gene-coding region was the LSC, which was 82,432 bp, 82,294 bp, 81,923 bp, 81,894 bp, 82,177 bp and 82,427 bp, respectively. The SSC regions were 19,273 bp, 19,225 bp, 17,872 bp, 17,844 bp, 18,244 bp and 17,677 bp in size, respectively. The IR (IRa, IRb) regions were 31,118 bp, 31,119 bp, 31,343 bp, 31,356 bp, 31,187 bp and 31,410 bp in size, respectively. We also compared the cp genomes of *A. carmichaelii* and *C. chinensis* with those of the six sequenced species, and found that their sizes were highly divergent (Table 1). In addition, we found that the total cp genome size of *P. chinensis* var. *kissii* was only 203 bp different from that of *Amomum compactum* in different families [38].

**Table 1:** Comparison of general characteristics of the cp genomes of the eight Ranunculaceae species

Type	*P. chinensis*	*P. chinensis* var. *kissii*	*P. cernua* f. *plumbea*	*P. dahurica*	*P. turczaninovii*	*P. cernua*	*A. carmichaelii*	*C. chinensis*
Size (bp)	163,851	163,756	162,481	162,450	162,795	162,924	155,737	155,484
GC content (%)	37.14	37.16	37.42	37.42	37.40	37.35	38.10	38.17
LSC length (bp)	82,342	82,294	81,923	81,894	82,177	82,427	86,330	84,585
SSC length (bp)	19,272	19,224	17,871	17,843	18,243	17,676	17,021	17,383
IR length (bp)	31,118	31,119	31,343	31,356	31,187	31,410	26,193	26,758
Gene number	134	134	134	134	134	134	112	128
Gene number in IR regions	46	46	46	46	46	46	36	36
Protein-coding gene number	90	90	90	90	90	90	78	92
rRNA gene number	8	8	8	8	8	8	4	8
tRNA gene number	36	36	36	36	36	36	30	28

A total of 134 genes were observed in each *Pulsatilla* cp genome, which was the same as that observed in the earliest differentiated group of flowering plants, *Amborella trichopoda* [39]. All genes, including 36 tRNAs, eight rRNAs and 90 protein-coding genes, were consistent in number. The six species of *Pulsatilla* all had 14 tRNAs and all eight rRNAs located in the IR region. Based on short read sequencing, we found that the LSC regions of the six *Pulsatilla* cp genomes were very similar in size, indicating that the evolution of the cp genes from the six *Pulsatilla* species was highly conserved (Table 1).

**Fig. 1:**
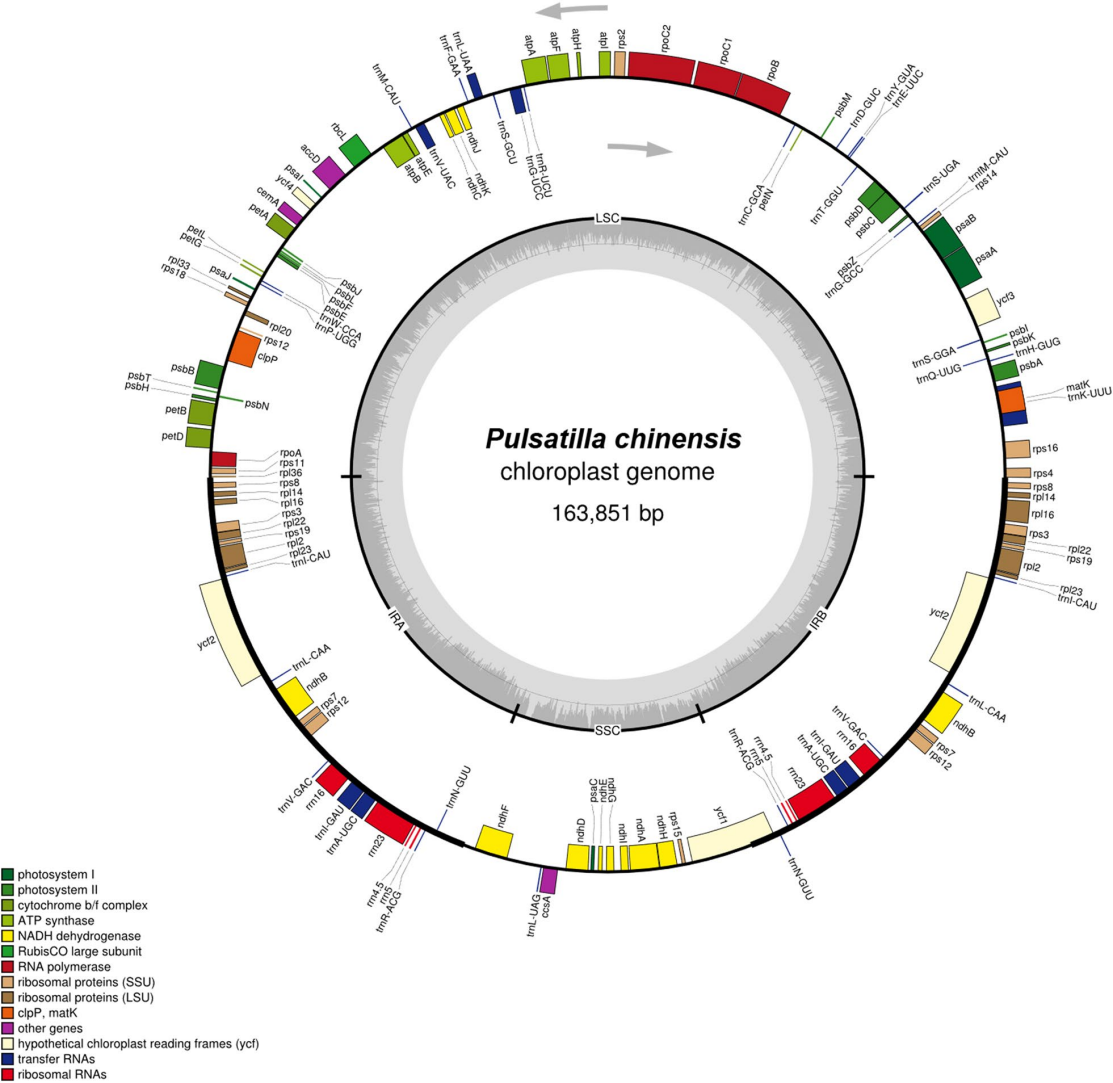
Circular gene map of *P. chinensis.* Genes on the outside circle are transcribed counterclockwise, while genes on the inside circle are transcribed clockwise. *LSC* large single copy, *SSC* small single copy, *IRa* inverted repeat a, *IRb* inverted repeat b

The relative locations and sizes of different genes were mostly similar in the six species of *Pulsatilla*. For example, the *matK* genes of the six species were all located in the *trnK*-*UUU* gene region. The *rpl16* gene of *P. chinensis* in the IRb region was always larger than that in the IRa region. On the other hand, the *rpl16* gene of *P. chinensis* var. *kissii* in the IRa region was larger than of the one in the IRb region. The *rpl16* genes in the IRa and IRb regions of *P. cernua* were larger than the other five *Pulsatilla* species, which can be used as a species identification characteristic. In the *rpl16* gene characteristic, *P. dahurica* and *P. cernua* were identical, although the total genome size of the latter was 31 bp longer than that of the former. The only difference between *P. dahurica* and *P. turczaninovii* is was the *rps12* gene in the IRa region, with the latter having a much smaller size. This was a unique characteristic of *P. turczaninovii*, compared with all five other *Pulsatilla* species. Although the cp genes of *P. dahurica* and *P. cernua* were mostly similar. The cp genome of the former was 474 bp longer that the latter. The *ycf15* gene, which is present in angiosperms, was missing in the cp genomes of *Pulsatilla* and *Illicium* (Schisandraceae) [40]. Due to these various differences, genes such as *rpl16* and *rps12* can be used to develop molecular markers for the differentiation of these species (Fig. 1, Additional file 1: Figure S1, Additional file 2: Figure S2, Additional file 3: Figure S3, Additional file 4: Figure S4 and Additional file 5: Figure S5).

Among the cp genes of the six *Pulsatilla* species, the two largest gene families were involved in photosynthesis and self-replication (Table 2). There were six genes coding for subunits of the ATP synthase (*atpA*, *atpB*, *atpE*, *atpF*, *atpH* and *atpI*) and 12 genes coding for subunits of NADH dehydrogenase (*ndhA*, *ndhB*, *ndhC*, *ndhD*, *ndhE*, *ndhF*, *ndhG*, *ndhH*, *ndhI*, *ndhJ* and *ndhK*), which contained two *ndhB* genes. Five (*psaA*, *psaB*, *psaC*, *psaI* and *psaJ*) and 15 genes (*psbA*, *psbB*, *psbC*, *psbD*, *psbE*, *psbF*, *psbH*, *psbI*, *psbJ*, *psbK*, *psbL*, *psbM*, *psbN*, *psbT* and *psbZ*) coded for the subunits of photosystem I and photosystem II, respectively. This was consistent with the number of genes identified in *Acer miaotaiense* [34]. The function of five of the 134 genes, including *ycf1*, *ycf3*, *ycf4*, and two copies of *ycf2*, were not known.

**Table 2:** List of the genes in the cp genomes of six species of *Pulsatilla*

Gene category	Gene group	Gene name
Photosynthesis	Subunits of ATP synthase (6)	*atpA*, *atpB*, *atpE*, *atpF*^a^, *atpH*, *atpI*
	Subunits of NADH dehydrogenase (12)	*ndhA*^a^, *ndhB*^a^ (x2), *ndhC*, *ndhD*, *ndhE*, *ndhF*, *ndhG*, *ndhH*, *ndhI*, *ndhJ*, *ndhK*
	Subunits of cytochrome (6)	*petA*, *petB*^a^, *petD*^a^, *petG*, *petL*, *petN*
	Subunits of photosystem I (5)	*psaA*, *psaB*, *psaC*, *psaI*, *psaJ*
	Subunits of photosystem II (15)	*psbA*, *psbB*, *psbC*, *psbD*, *psbE*, *psbF*, *psbH*, *psbI*, *psbJ*, *psbK*, *psbL*, *psbM*, *psbN*, *psbT*, *psbZ*
Other genes	Subunit of rubisco (1)	*rbcL*
	Subunit of Acetyl-CoA-carboxylase (1)	*accD*
	c-type cytochrome synthesis gene (1)	*ccsA*
	Envelop membrane protein (1)	*cemA*
	Protease (1)	*clpP*^b^
	Maturase (1)	*matK*
Self-replication	Large subunit of ribosome (13)	*rpl2*^a^ (x2), *rpl14* (x2), *rpl16*^a^ (x2), *rpl20*, *rpl22* (x2), *rpl23* (x2) *rpl33*, *rpl36*
	DNA dependent RNA polymerase (4)	*rpoA*, *rpoB*, *rpoC1*^a^, *rpoC2*
	Small subunit of ribosome (18)	*rps2*, *rps3* (x2), *rps4*, *rps7* (x2), *rps8* (x2), *rps11*, *rps12*^a^ (x3), *rps14*, *rps15*, *rps16*^a^, *rps18*, *rps19* (x2)
	rRNA Genes (8)	*rrn4.5* (x2), *rrn5* (x2), *rrn16* (x2), *rrn23* (x2)
	tRNA Genes (36)	*trnA*-*UGC* (x2), *trnC*-*GCA*, *trnD*-*GUC*, *trnE*-*UUC*, *trnF*-*GAA*, *trnG*-*GCC*, *trnG*-*UCC*, *trnH*-*GUG*, *trnI*-*CAU* (x2), *trnI*-*GAU* (x2), *trnK*-*UUU*, *trnL*-*CAA* (x2), *trnL*-*UAA*, *trnL*-*UAG*, *trnfM*-*CAU*, *trnM*-*CAU*, *trnN*-*GUU* (x2), *trnP*-*UGG*, *trnQ*-*UUG*, *trnR*-*ACG* (x2), *trnR*-*UCU*, *trnS*-*GCU*, *trnS*-*GGA*, *trnS*-*UGA*, *trnT*-*GGU*, *trnV*-*GAC* (x2), *trnV*-*UAC*, *trnW*-*CCA*, *trnY*-*GUA*
Unknown function	Conserved open reading frames (5)	*ycf 1*, *ycf 2* (x2), *ycf 3*^b^, *ycf4*

Numbers in brackets behind the name of gene group refer to the number of repetitive genes

^a^Contains one intron

^b^Contains two introns

Introns play an important role in regulating gene expression. Recent studies have found that many introns can enhance the expression of exogenous genes at specific times and locations, with implications for agronomic trait improvement [41]. The protein coding genes of all six *Pulsatilla* species contained the same number of introns. *rps16*, *rpoC1*, *atpF*, *petB*, *petD*, *rpl16*, *rpl2*, *ndhB*, *rps12*, and *ndhA* all display one intron, while *ycf*3 and *clpP* have two introns (Additional file 6: Table S1).

### Repeat sequence analysis

#### SSR analysis

Microsatellite markers, also known as SSR markers, are PCR-based DNA molecular markers [42]. Because of the characteristics of neutral markers, the highly variable numbers of repeats and the relative conservation of flanking sequences of SSRs, they have been widely utilized in genotyping. SSR marker are also easy to design and have high repeatability and codominant inheritance among alleles, making them the best choice for evaluating the genetic diversity of crop species [43–45].

**Fig. 2:**
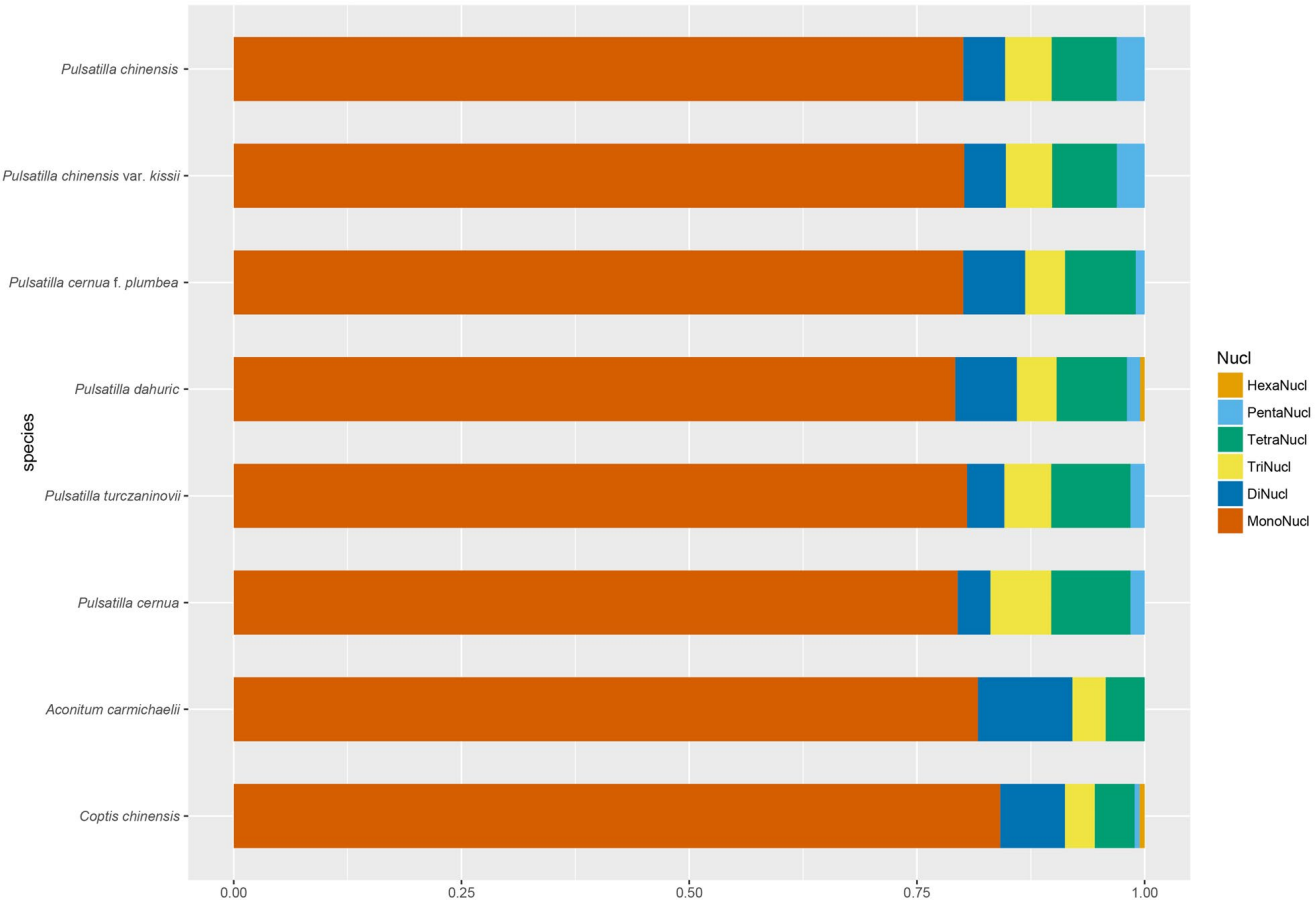
SSRs in the cp genomes of eight species in Ranunculaceae. MonoNucl represents mononucleotide repeats, DiNucl represents dinucleotide repeats, TriNucl represents trinucleotide repeats, TetraNucl represents tetranucleotide repeats, PentaNucl represents pentanucleotide repeats, and HexaNucl represents hexalnucleotide repeats

The cp genomes of *P. chinensis*, *P. chinensis* var. *kissii*, *P. cernua* f. *plumbea*, *P. dahurica*, *P. turczaninovii*, and *P. cernua*, contained 196, 197, 207, 208, 196, and 196 SSRs, respectively (Table 3). This is significantly different from that observed with *Arctium*, which had far less SSRs [46]. The length of SSRs were 116 bp, 116 bp, 114 bp, 114 bp, 126 bp and 176 bp, respectively. The number of SSRs in the formation of compound microsatellites was 48, 48, 55, 57, 46 and 47, respectively. There were six types of SSRs in *P. dahurica*, including mononucleotide, dinucleotide, trinucleotide, tetranucleotide, pentanucleotide, and hexanucleotide, other five species of *Pulsatilla* contained five types of SSRs other than hexanucleotide (Fig. 2). The six species of *Pulsatilla* contained mostly A/T SSRs, with 149, 150, 157, 156, 150 and 147, respectively. They accounted for 76.00%, 76.14%, 75.85%, 75.00%, 76.53%, 75.00% of all SSR nucleotides, respectively (Additional file 7: Table S2). The AT content was relatively high, which may be related to the relative stability of AT and GC base pairs [47]. In SSRs, only *P. turczaninovii* contained seven C/G repeats, while other *Pulsatilla* contained eight C/G, which can be used as a distinguishing characteristic. In addition, the AT content of *P. cernua* f. *plumbea* and *P. dahurica* was very high (6.76% and 6.73%, respectively), which was significantly different from those of other *Pulsatilla*. The SSRs of the six species of *Pulsatilla* were also compared with those of *A. carmichaelii* and *C. chinensis*. The SSRs of *C. chinensis* also contained hexanucleotide SSRs, which were found in *P. dahurica*. However, *A. carmichaelii* did not contain pentanucleotide or hexanucleotide SSRs. Compared with other types of nucleotide repeats, the six species of *Pulsatilla* contained more mononucleotides repeats (Additional file 7: Table S2). The SSRs of several species in Ranunculaceae were similar, making SSR markers a tenable approach.

**Table 3:** SSRs identified in the cp genomes of the six *Pulsatilla* species

Unit size	*P. chinensis*	*P. chinensis* var. *kissii*	*P. cernua* f. *plumbea*	*P. dahurica*	*P. turczaninovii*	*P. cernua*
Mononucleotide	157	158	165	164	157	155
Dinucleotide	9	9	14	14	8	7
Trinucleotide	10	10	10	10	11	14
Tetranucleotide	14	14	16	16	17	17
Pentanucleotide	6	6	2	3	3	3
Hexanucleotide	0	0	0	1	0	0

Taking *P. chinensis* as the representative species, the distribution of palindromic-type SSR (p-type SSR) was analyzed (Additional file 8: Table S3). The repeat sequences were mainly distributed in the non-coding sequences (CNS), intergenic spacers, and intron regions. Some were found in the coding regions of certain genes, including *matK*, *psbC*, *rpoB*, *rpoC2*, *rps2*, *atpA*, *ndhJ*, *atpB*, *accD*, and others. The other five *Pulsatilla* species were similar to *P. chinensis* in terms of p-type SSR (Additional file 9: Table S4, Additional file 10: Table S5, Additional file 11: Table S6, Additional file 12: Table S7 and Additional file 13: Table S8).

#### Large repeat analysis

Many repeats are present in gene deserts, although whole-genome sequencing has shown that they can occur in functional regions as well [48]. Repeat of 30 bases or more are typically considered large repeats. Forty-nine (*P. chinensis*), 49 (*P. chinensis* var. *kissii*), 38 (*P. cernua* f. *plumbea*), 38 (*P. dahurica*), 49 (*P. turczaninovii*) and 49 (*P. cernua*) pairs of large repeat sequences were found in the six *Pulsatilla* cp genomes, with sequence identity exceeding 90%. The repeats from *P. chinensis* and *P. chinensis* var. *kissii* ranged from 30 to 59 bp in length, and in *P. cernua* f. *plumbea*, *P. dahurica*, *P. turczaninovii* and *P. cernua*, the repeats ranged from 30 to 52 bp in length. A total of 26, 39, 32, 37, 32 and 43 large repeats were located in the genic regions of the six *Pulsatilla* species, respectively (Additional file 14: Table S9, Additional file 15: Table S10, Additional file 16: Table S11, Additional file 17: Table S12, Additional file 18: Table S13 and Additional file 19: Table S14).

### Analysis of the LSC, SSC, and IR border regions

**Fig. 3:**
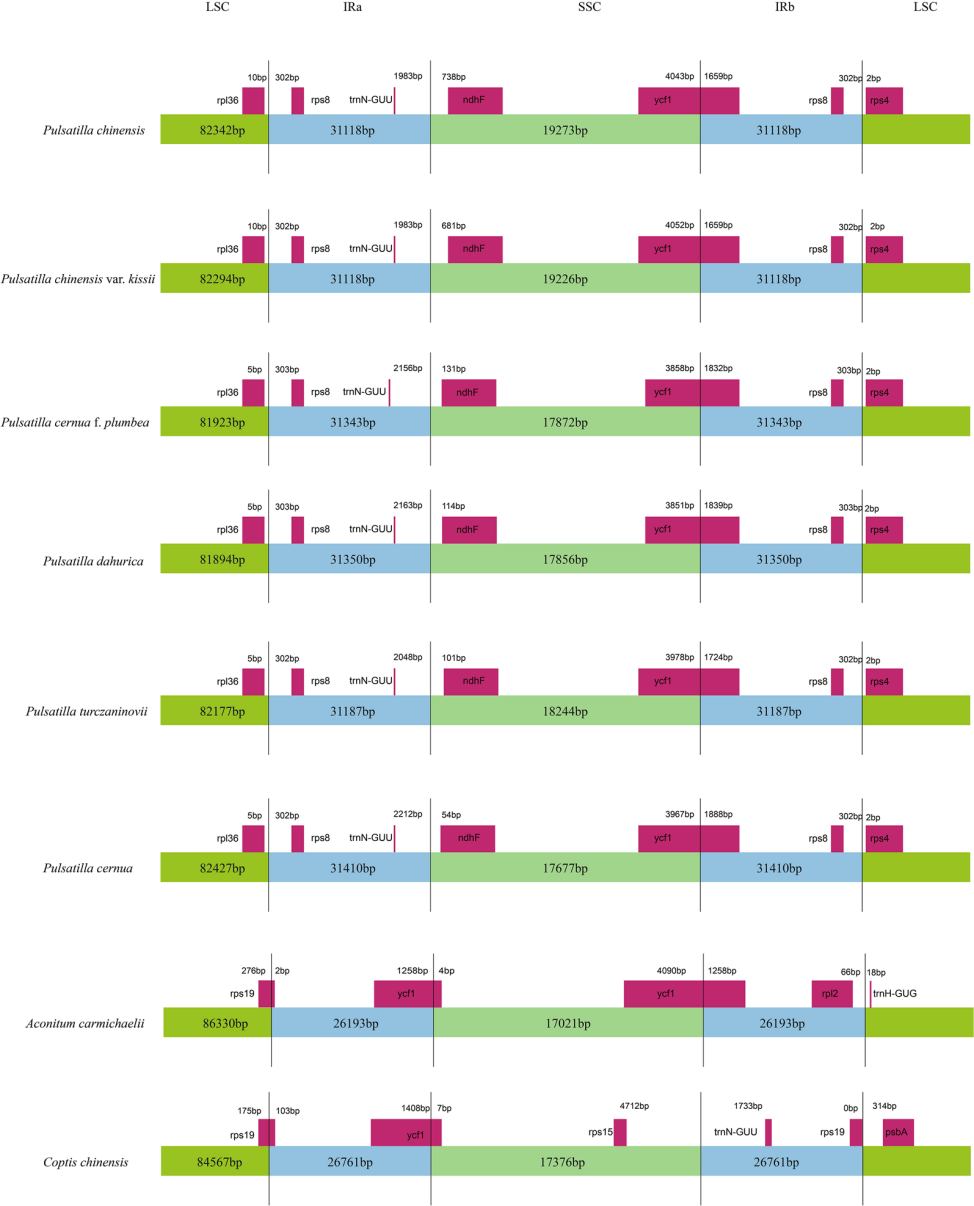
Comparison of the LSC, SSC, and IR border regions among the eight Ranunculaceae cp genomes. The number above gene features represents the distance between gene ends and the borders sites

Contraction and expansion of the IR regions may have caused the differences in cp genome size in plant groups [49]. By comparing the IR boundary characteristics of *P. chinensis*, *P. chinensis* var. *kissii*, *P. cernua* f. *plumbea*, *P. dahurica*, *P. turczaninovii*, *P. cernua*, *A. carmichaelii* and *C. chinensis*, we found that the length of IR region of the eight species ranged from 26,193 to 31,410 bp, while that of the six *Pulsatilla* species had a relatively narrower range between 31,118 and 31,410 bp (Fig. 3). Among the six species of *Pulsatilla*, the *ndhF* gene was located downstream of the IRa/LSC border, which was the same as *Arctium lappa* and *Magnolia grandiflora* [46, 50] but different from most angiosperms [51]. The *ycf1* gene of the six *Pulsatilla* species was located across the IRb and SSC region boarder, which was the same as that observed in *A. carmichaelii*. The IRa/SSC and IRa/LSC boarders in *A. carmichaelii* and *C. chinensis* were located inside the *ycf1* and *rps19* genes. The *rps19* genes of both species were 278 bp in length, whereas the length of *ycf*1 varied, which was not observed in the six *Pulsatilla* species. The *trnN*-*GUU* genes were in the IRa region in the six *Pulsatilla* species and the distance between *trnN*-*GUU* genes and the IRa/SSC border were not all the same in the six *Pulsatilla* species. Among six *Pulsatilla* species, the distances in *P. chinensis* and *P. chinensis* var. *kissii* were the smallest (1983 bp), while that in *P. cernua* was the largest (2212 bp). However, the *trnN*-*GUU* gene of *C. chinensis* was in the IRb region. These results showed that the IR regions of six *Pulsatilla* species were highly conserved, but were quite different from those of *A. carmichaelii* and *C. chinensis*, which are from different genera of the same family. The length of IR region is not constant during the evolution of cp genomes. Some studies have shown that the amplification of reverse repeats of the buckwheat cp genome may be a result of reverse transcription [52].

#### Nucleotide diversity analysis

**Fig. 4:**
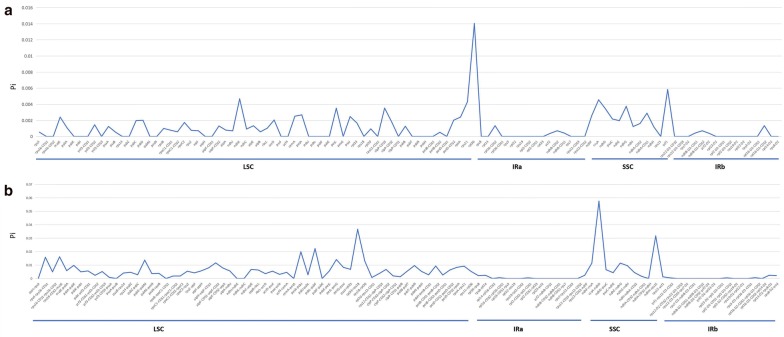
Comparative analysis of the nucleotide variability by Pi values of the six *Pulsatilla* species (**a** coding region, **b** non-coding region)

Both the coding and non-coding regions of cp genome sequences are highly variable, these sequences can thus be utilized to determine the phylogenetic relationship between species and genera. In total, 108 coding genes (Fig. 4a) and 105 non-coding genes (Fig. 4b) were found in the six *Pulsatilla* cp genomes. The Pi values of coding genes ranged between 0 and 0.0140351, while those of the non-coding regions were between 0 and 0.0575316. In both cases, most Pi values were zero (Additional file 20: Table S15). Overall, the non-coding regions were more conserved than the coding regions. The two most variable loci were *rpl36* (Pi = 0.0140351) and *ccsA*-*ndhD* (Pi = 0.0575316), which are located in the LSC and SSC region, respectively. This is consistent with earlier results that the IR region is generally more conserved than the LSC and SSC regions [53, 54].

#### Phylogenetic analysis

**Fig. 5:**
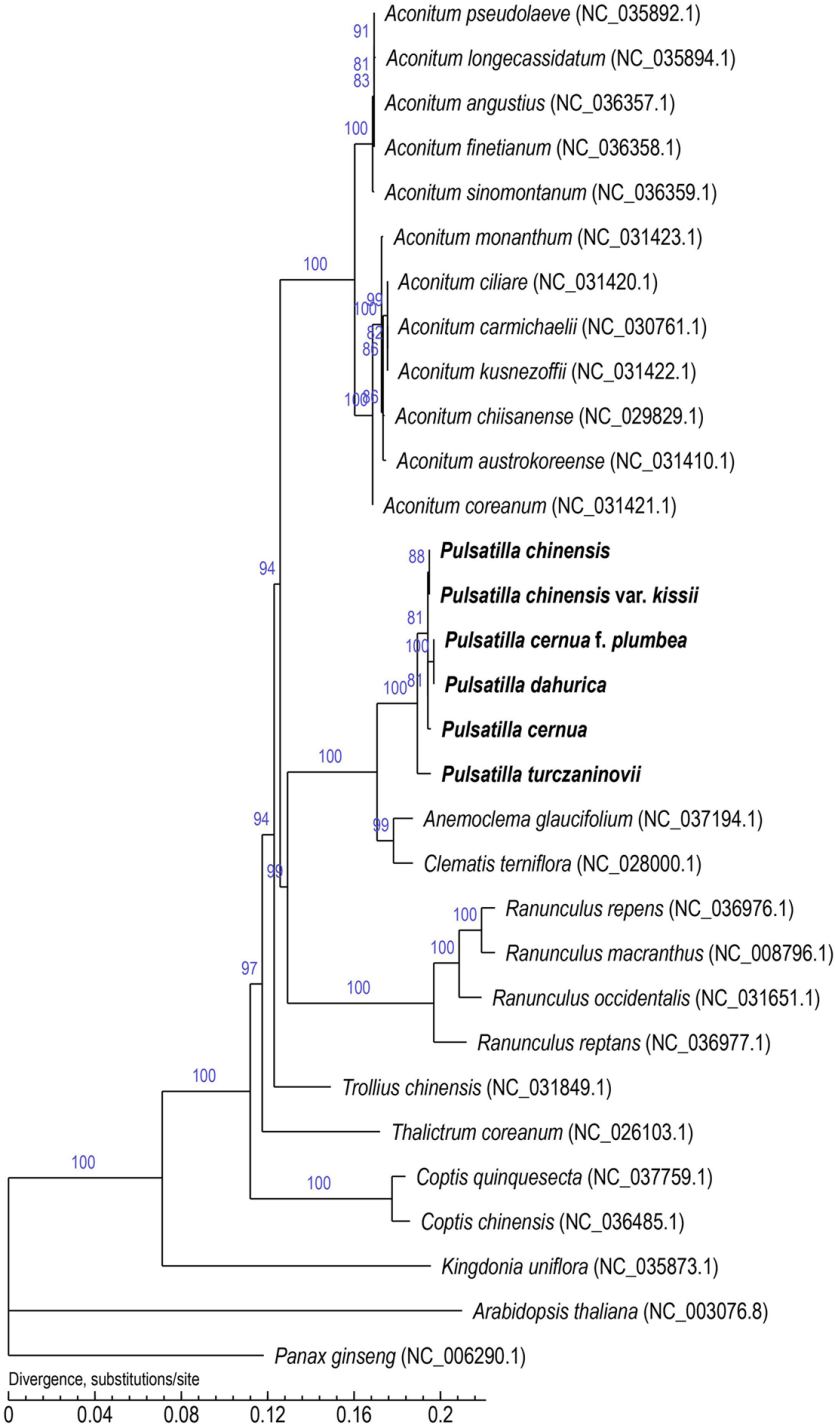
Molecular phylogenetic tree of 31 species based on whole cp genome SNPs. The tree was constructed using the maximum likelihood method in PhyML v3.0 with 1000 bootstrap replications

The variation of angiosperm cp genomes is much higher than can be monitored with a single DNA barcode sequence. This sequence variability can be utilized for studying the phylogenetic relationships among closely related species, and is also valuable for species identification. The cp genome has also been suggested as a super barcode [55]. In our previous study, DNA barcodes were used to identify eight *P*. Miller species present in different medicinal materials. These earlier results showed that ITS2 and *psb*A-*trn*H sequences can identify these species accurately, while *rbc*L and *mat*K sequences cannot [4, 56]. To determine the phylogenetic position of *P.* Miller in Ranunculaceae, a phylogenetic tree was constructed based on SNPs of the whole cp genomes of the six *Pulsatilla* species, 23 species from the other genera in Ranunculaceae, and two species of the outgroups (Fig. 5). In the resulting phylogenetic tree, 13 of 28 nodes exhibited bootstrap values of 100% and seven had bootstrap values of 90%. The six *Pulsatilla* species clustered closely together into one branch. This result is consistent with earlier phylogenetic analyses based on DNA molecular identification of several mixed *Pulsatilla* samples [57].

*Pulsatilla chinensis* and *P. chinensis* var. *kissii* clustered into one branch, implying that they are very closely related. *P. chinensis* var. *kissii* is also recorded as a variety of *P. chinensis* in both *Flora Reipubicae Popularis Sinicae* and *Herbaceous Flora of Northeast China* [2, 58], which was further validated by our clustering results. *P. cernua* f. *plumbea* and *P. dahurica* were clustered into one branch. In fact, *P. cernua* f. *plumbea* is considered a forma of *P. cernua* [59], but our results showed that they are relatively distant within the *Pulsatilla* branch. *P. turczaninovii* was relatively far from other *Pulsatilla* species. This indicates that *P. turczaninovii* and other *Pulsatilla* species were relatively distantly related. In reality, *P. turczaninovii* is also recorded as a separate species in some literature [2, 58, 60]. Among these six species, only *P. chinensis* is included in the *Chinese Pharmacopoeia* as a original plant, but other *Pulsatilla* members also clustered with *P. chinensis* in our tree. Whether other *Pulsatilla* species can replace *P. chinensis* requires further verification.

In our phylogenetic tree, *Anemoclema glaucifolium* and *Clematis terniflora* were clustered in one clade, and they both were closely related. At the same time, they both were clustered in one large clade with six species of *Pulsatilla*. This indicates that *Pulsatilla* species were more closely related to both than was previously thought. *Ranunculus repens*, *R. macranthus*, *R. occidentalis* and *R. reptans* gathered in one clade, with *R. repens* and *R. macranthus* clustered into one small branch. *Trollius chinensis* and *Thalictrum coreanum* were also closely related. *C. quinquesecta* and *C. chinensis* were gathered in one clade. In addition, *Kingdonia uniflora* was clustered in a single branch, which indicated that it was distantly related to other Ranunculaceae species. *Aconitum* L. were clustered into one branch, with *A. pseudolaeve*, *A. longecassidatum*, *A. angustius*, *A. finetianum* and *A. sinomontanum* gathered in one small clade. Previous studies on *A. longecassidatum* and *A. pseudolaeve* have shown that they have highly conserved cp genome structure [61], which fits with our results. *A. monanthum*, *A. ciliare*, *A. carmichaelii*, *A. kusnezoffii*, *A. chiisanense*, *A. austrokoreense* and *A. coreanum* gathered in one small clade, which was also consistent with previous research results [62]. *Arabidopsis thaliana* and *Panax ginseng* were located at the bottom of the phylogenetic tree, and clustered into one branch. The phylogenetic relationship of cp genomes in Ranunculaceae was analyzed by SNPs sequence, which indicated the cp genomes in Ranunculaceae was relatively conserved, and the six species of *Pulsatilla* were closely related.

**Fig. 6:**
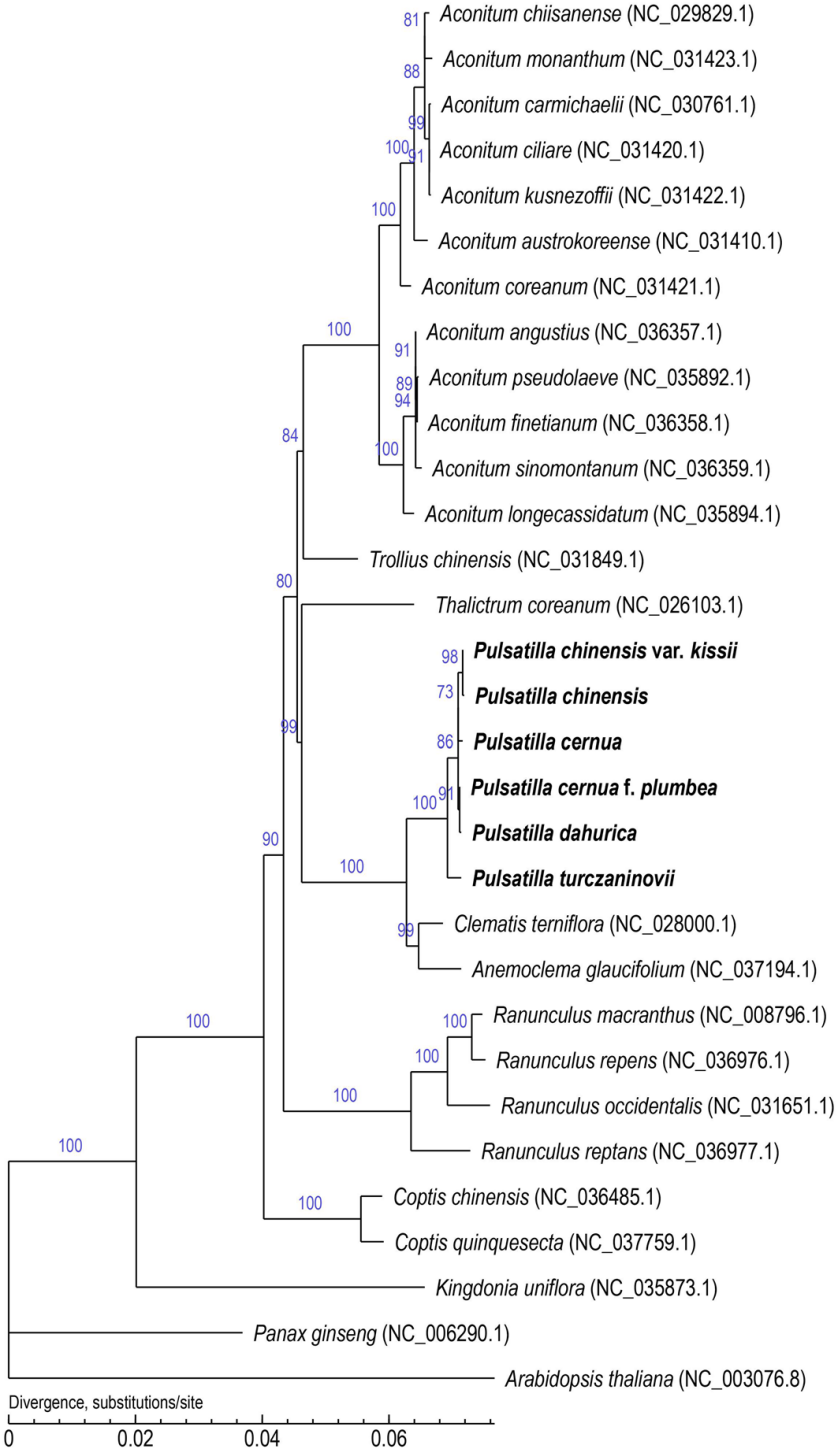
Molecular phylogenetic tree of 31 species based on 51 shared chloroplastic protein-coding. The tree was constructed via a maximum likelihood analysis using PhyML v3.0 with 1000 bootstrap replications

Another phylogenetic tree of 31 species was constructed based on 51 shared protein-coding sequences, with protein similarity threshold set at > 40% (Fig. 6). Twelve of the 28 nodes had bootstrap values of 100% and nine nodes had bootstrap values ≥ 90%. The six *Pulsatilla* species formed one closely related clade. Overall, the results of the two methods of building trees were similar, with *P. chinensis*, *P. chinensis* var. *kissii*, *P. cernua* f. *plumbea*, and *P. dahurica* clustering into one small clade. *P. turczaninovii* was once again far away from other *Pulsatilla*. Twelve species of *Aconitum* were grouped into one branch. Two species of *Coptis* and four species of *Ranunculus* were also clustered into one branch. *C. terniflora* and *A. glaucifolium* composed one closely related clade. *T. chinensis*, *T. coreanum* and *K. uniflora* were clustered into different branches. *P. ginseng* and *A. thaliana* were also located at the bottom of the phylogenetic tree, and clustered into one branch. Ranunculaceae diverged early during eudicot evolution and is increasingly being used as a model for the study of plant evolution. Previous studies have shown that two radiation waves occurred early on in Ranunculaceae evolution, generating most extant tribes and genera [63]. Through the construction of two phylogenetic trees, the position of *Pulsatilla* in Ranunculaceae was elucidated, with implications for future evolutionary analysis.

## Conclusion

Analysis of the cp genome sequences of six *Pulsatilla* species showed that they had very similar genome sizes. Comparison to *A. carmichaelii* and *C. chinensis* in Ranunculaceae revealed differences in genome sizes. For example, the size of annotated cp genes in the six *Pulsatilla* was different, particularly *rpl16* and *rps12*. These differences may be useful for marker development and phylogenetic analysis. In addition, *P. dahurica* contained six types of SSRs, while the other five *Pulsatilla* species had only five SSR types (with no hexanucleotides identified). Thirty-eight to 49 pairs of large repeats were found in the six *Pulsatilla* cp genomes, which are valuable for marker development and phylogenetic analysis. In addition, the size of IR regions was conserved among *Pulsatilla* species but different from that of *A. carmichaelii* and *C. chinensis* in Ranunculaceae. We also analyzed the nucleotide diversity of 108 genes and 105 non-coding regions, and *rpl36* and *ccsA*-*ndhD* were the most variable, which are potentially suitable for marker design. The phylogenetic analysis was conducted based on SNPs of the whole cp genome and 51 shared chloroplastic protein-coding, and the position of six *Pulsatilla* species in Ranunculaceae was determined. These two phylogenetic trees will provide a reference for studying the evolutionary history of Ranunculaceae.

## Supplementary information

**Additional file 1: Figure S1.** Circular gene map of *P. chinensis* var. *kissii*.

**Additional file 2: Figure S2.** Circular gene map of *P. cernua* f. *plumbea*.

**Additional file 3: Figure S3.** Circular gene map of *P. dahurica*.

**Additional file 4: Figure S4.** Circular gene map of *P. turczaninovii*.

**Additional file 5: Figure S5.** Circular gene map of *P. cernua*.

**Additional file 6: Table S1.** Location and length of intron-containing cp genes within the six *Pulsatilla* species.

**Additional file 7: Table S2.** Type and abundance of different SSRs in six species of *Pulsatilla*.

**Additional file 8: Table S3.** SSRs distribution of the *P. chinensis* cp genome.

**Additional file 9: Table S4.** SSRs distribution of the *P. chinensis* var. *kissii* cp genome.

**Additional file 10: Table S5.** SSRs distribution of the *P. cernua* f. *plumbea* cp genome.

**Additional file 11: Table S6.** SSRs distribution of the *P. dahurica* cp genome.

**Additional file 12: Table S7.** SSRs distribution of the *P. turczaninovii* cp genome.

**Additional file 13: Table S8.** SSRs distribution of the *P. cernua* cp genome.

**Additional file 14: Table S9.** Large repeats identified in the *P. chinensis* cp genome.

**Additional file 15: Table S10.** Large repeats identified in the *P. chinensis* var. *kissii* cp genome.

**Additional file 16: Table S11.** Large repeats identified in the *P. cernua* f. *plumbea* cp genome.

**Additional file 17: Table S12.** Large repeats identified in the *P. dahurica* cp genome.

**Additional file 18: Table S13.** Large repeats identified in the *P. turczaninovii* cp genome.

**Additional file 19: Table S14.** Large repeats identified in the *P. cernua* cp genome.

**Additional file 20: Table S15.** Pi values of the coding and no-coding regions in the six *Pulsatilla* cp genomes.

## Data Availability

All data generated or analyzed during the course of this study are included in this document or obtained from the appropriate author(s) at reasonable request.

## Abbreviations

cp: chloroplast
LSC: large single copy
SSC: small single copy
IR: inverted repeat
tRNA: transfer RNA
rRNA: ribosomal RNA
