# Supplementary material for: Comparative analysis of the complete chloroplast genome sequences of six species of Pulsatilla Miller, Ranunculaceae
Source: Chin Med. 2019 Nov 28;14:53. doi: 10.1186/s13020-019-0274-5 (PMC6883693; doi:10.1186/s13020-019-0274-5)
Supplement: Supplementary file 2 — Additional file 2: Figure S2. Circular gene map of P. cernua f. plumbea. [file 13020_2019_274_MOESM2_ESM.docx]

**
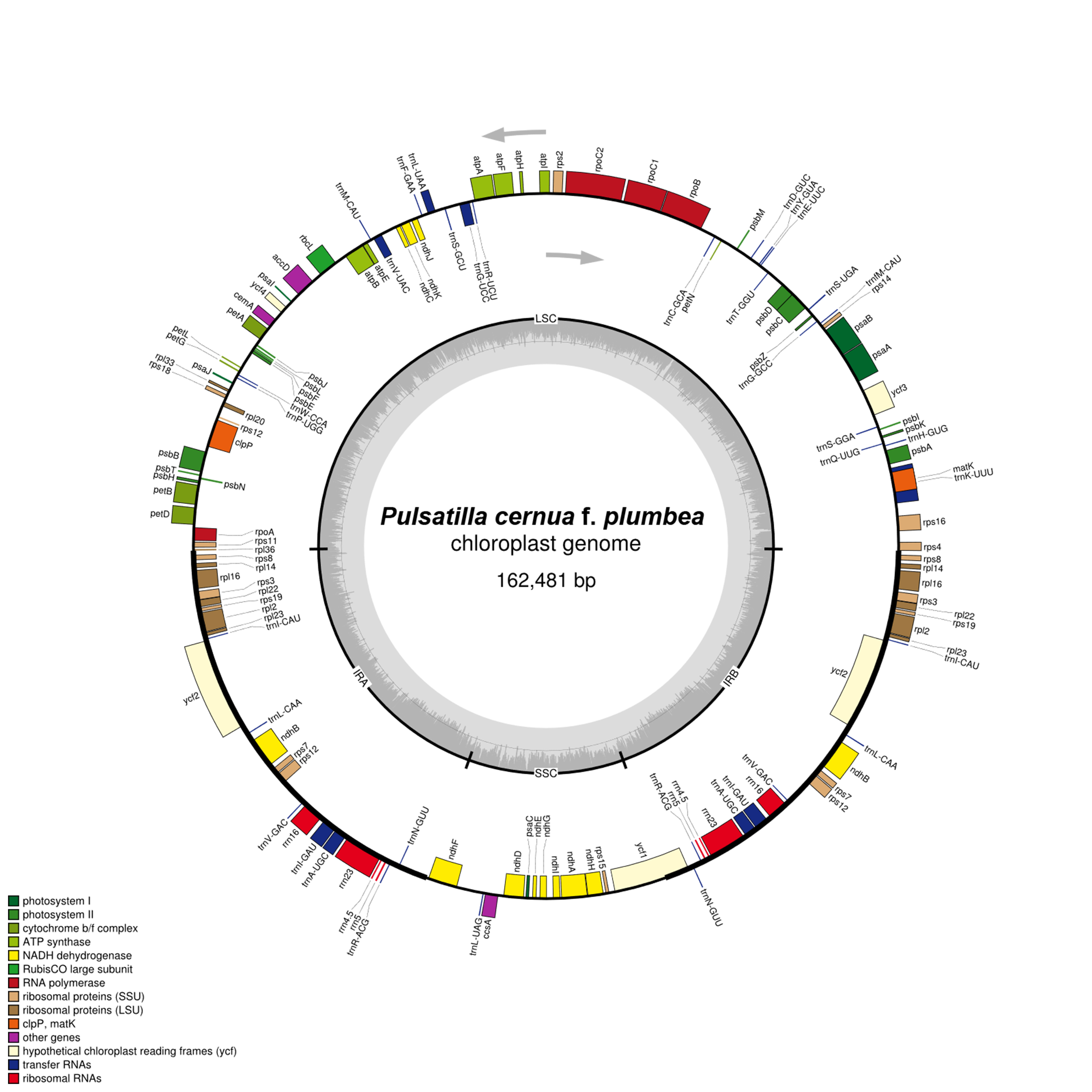
Figure S2** Circular gene map of *P. cernua* f. *plumbea*. Genes on the outside circle are transcribed counterclockwise, while genes on the inside circle are transcribed clockwise. LSC, large single copy; SSC, small single copy; IRa, inverted repeat a; IRb, inverted repeat b
