# Supplementary material for: Comparative analysis of the complete chloroplast genome sequences of six species of Pulsatilla Miller, Ranunculaceae
Source: Chin Med. 2019 Nov 28;14:53. doi: 10.1186/s13020-019-0274-5 (PMC6883693; doi:10.1186/s13020-019-0274-5)
Supplement: Supplementary file 6 — Additional file 6: Table S1. Location and length of intron-containing cp genes within the six Pulsatilla species. [file 13020_2019_274_MOESM6_ESM.docx]

**Table S1 Location and length of intron-containing cp genes within the six *Pulsatilla* species**

|  | **Gene** | **Location** | **ExonI (bp)** | **IntronI (bp)** | **ExonII (bp)** | **IntronII (bp)** | **ExonIII (bp)** |
| --- | --- | --- | --- | --- | --- | --- | --- |
| *P. chinensis* | *rps16* | LSC | 39 | 884 | 193 |  |  |
|  | *ycf3* | LSC | 123 | 726 | 229 | 765 | 152 |
|  | *rpoC1* | LSC | 429 | 756 | 1609 |  |  |
|  | *atpF* | LSC | 144 | 789 | 409 |  |  |
|  | *clpP* | LSC | 245 | 756 | 288 | 792 | 70 |
|  | *petB* | LSC | 5 | 857 | 641 |  |  |
|  | *petD* | LSC | 7 | 745 | 495 |  |  |
|  | *rpl16* | IR | 398 | 991 | 8 |  |  |
|  | *rpl2* | IR | 433 | 664 | 390 |  |  |
|  | *ndhB* | IR | 755 | 707 | 776 |  |  |
|  | *rps12** | LSC | 113 | - | 231 | 542 | 25 |
|  | *ndhA* | SSC | 538 | 873 | 552 |  |  |
| *P. chinensis* var*. kissii* | *rps16* | LSC | 39 | 884 | 193 |  |  |
|  | *ycf3* | LSC | 123 | 726 | 229 | 765 | 152 |
|  | *rpoC1* | LSC | 429 | 756 | 1609 |  |  |
|  | *atpF* | LSC | 144 | 783 | 409 |  |  |
|  | *clpP* | LSC | 245 | 756 | 288 | 792 | 70 |
|  | *petB* | LSC | 5 | 857 | 641 |  |  |
|  | *petD* | LSC | 7 | 745 | 495 |  |  |
|  | *rpl16* | IR | 398 | 991 | 8 |  |  |
|  | *rpl2* | IR | 433 | 664 | 390 |  |  |
|  | *ndhB* | IR | 755 | 707 | 776 |  |  |
|  | *rps12** | LSC | 113 | - | 231 | 542 | 25 |
|  | *ndhA* | SSC | 538 | 873 | 552 |  |  |
| *P. cernua* f. *plumbea* | *rps16* | LSC | 39 | 886 | 193 |  |  |
|  | *ycf3* | LSC | 123 | 726 | 229 | 769 | 152 |
|  | *rpoC1* | LSC | 429 | 756 | 1615 |  |  |
|  | *atpF* | LSC | 144 | 784 | 409 |  |  |
|  | *clpP* | LSC | 245 | 710 | 288 | 784 | 70 |
|  | *petB* | LSC | 5 | 817 | 641 |  |  |
|  | *petD* | LSC | 7 | 758 | 495 |  |  |
|  | *rpl16* | IR | 398 | 981 | 8 |  |  |
|  | *rpl2* | IR | 433 | 666 | 390 |  |  |
|  | *ndhB* | IR | 755 | 707 | 776 |  |  |
|  | *rps12** | LSC | 113 | - | 231 | 541 | 25 |
|  | *ndhA* | SSC | 538 | 848 | 552 |  |  |
| *P. dahurica* | *rps16* | LSC | 39 | 885 | 193 |  |  |
|  | *ycf3* | LSC | 123 | 726 | 229 | 769 | 152 |
|  | *rpoC1* | LSC | 429 | 756 | 1615 |  |  |
|  | *atpF* | LSC | 144 | 784 | 409 |  |  |
|  | *clpP* | LSC | 245 | 710 | 288 | 784 | 70 |
|  | *petB* | LSC | 5 | 817 | 641 |  |  |
|  | *petD* | LSC | 7 | 757 | 495 |  |  |
|  | *rpl16* | IR | 398 | 981 | 8 |  |  |
|  | *rpl2* | IR | 433 | 666 | 390 |  |  |
|  | *ndhB* | IR | 755 | 707 | 776 |  |  |
|  | *rps12** | LSC | 113 | - | 231 | 541 | 25 |
|  | *ndhA* | SSC | 538 | 848 | 552 |  |  |
| *P. turczaninovii* | *rps16* | LSC | 39 | 891 | 193 |  |  |
|  | *ycf3* | LSC | 123 | 726 | 229 | 773 | 152 |
|  | *rpoC1* | LSC | 429 | 760 | 1615 |  |  |
|  | *atpF* | LSC | 144 | 827 | 409 |  |  |
|  | *clpP* | LSC | 245 | 710 | 288 | 788 | 70 |
|  | *petB* | LSC | 5 | 798 | 641 |  |  |
|  | *petD* | LSC | 7 | 776 | 495 |  |  |
|  | *rpl16* | IR | 398 | 981 | 8 |  |  |
|  | *rpl2* | IR | 433 | 663 | 390 |  |  |
|  | *ndhB* | IR | 755 | 707 | 776 |  |  |
|  | *rps12** | LSC | 113 | - | 231 | 542 | 25 |
|  | *ndhA* | SSC | 538 | 855 | 552 |  |  |
| *P. cernua* | *rps16* | LSC | 38 | 888 | 193 |  |  |
|  | *ycf3* | LSC | 123 | 726 | 229 | 759 | 152 |
|  | *rpoC1* | LSC | 429 | 755 | 1615 |  |  |
|  | *atpF* | LSC | 144 | 878 | 409 |  |  |
|  | *clpP* | LSC | 245 | 720 | 288 | 783 | 70 |
|  | *petB* | LSC | 5 | 821 | 641 |  |  |
|  | *petD* | LSC | 7 | 756 | 495 |  |  |
|  | *rpl16* | IR | 398 | 977 | 8 |  |  |
|  | *rpl2* | IR | 433 | 662 | 390 |  |  |
|  | *ndhB* | IR | 755 | 707 | 776 |  |  |
|  | *rps12** | LSC | 113 | - | 231 | 547 | 25 |
|  | *ndhA* | SSC | 538 | 869 | 552 |  |  |

***rps12** gene is a trans-spliced gene with the two duplicated 3ˊend exons in the IR regions and a 5ˊend exon in the LSC region.**
