# Supplementary material for: Comparative analysis of the complete chloroplast genome sequences of six species of Pulsatilla Miller, Ranunculaceae
Source: Chin Med. 2019 Nov 28;14:53. doi: 10.1186/s13020-019-0274-5 (PMC6883693; doi:10.1186/s13020-019-0274-5)
Supplement: Supplementary file 7 — Additional file 7: Table S2. Type and abundance of different SSRs in six species of Pulsatilla. [file 13020_2019_274_MOESM7_ESM.docx]

**Table S2 Type and abundance of different SSRs in six species of *Pulsatilla***

|  | ***P. chinensis*** | | | ***P. chinensis*var. *kissii*** | | | |
| --- | --- | --- | --- | --- | --- | --- | --- |
|  | **SSR units** | **SSR abundances** | **Percent abundance (%)** | **SSR units** | **SSR abundances** | | **Percent abundance (%)** |
| Mononucleotide | A/T | 149 | 76.00 | A/T | 150 | | 76.14 |
|  | C/G | 8 | 4.08 | C/G | 8 | | 4.06 |
| Dinucleotide | AT/AT | 9 | 4.59 | AT/AT | 9 | | 4.57 |
| Trinucleotide | AAG/CTT | 1 | 0.51 | AAG/CTT | 1 | | 0.51 |
|  | AAT/ATT | 9 | 4.59 | AAT/ATT | 9 | | 4.57 |
| Tetranucleotide | AAAG/CTTT | 2 | 1.02 | AAAG/CTTT | 2 | | 1.02 |
|  | AAAT/ATTT | 2 | 1.02 | AAAT/ATTT | 2 | | 1.02 |
|  | AACG/CGTT | 1 | 0.51 | AACG/CGTT | 1 | | 0.51 |
|  | AACT/AGTT | 4 | 2.04 | AACT/AGTT | 4 | | 2.03 |
|  | AAGT/ACTT | 1 | 0.51 | AAGT/ACTT | 1 | | 0.51 |
|  | AATG/ATTC | 1 | 0.51 | AATG/ATTC | 1 | | 0.51 |
|  | AATT/AATT | 2 | 1.02 | AATT/AATT | 2 | | 1.02 |
|  | AGAT/ATCT | 1 | 0.51 | AGAT/ATCT | 1 | | 0.51 |
| pentanucleotide | AAAAT/ATTTT | 1 | 0.51 | AAAAT/ATTTT | 1 | | 0.51 |
|  | AAAGT/ACTTT | 1 | 0.51 | AAAGT/ACTTT | 1 | | 0.51 |
|  | AAATT/AATTT | 1 | 0.51 | AAATT/AATTT | 1 | | 0.51 |
|  | AAGAT/ATCTT | 1 | 0.51 | AAGAT/ATCTT | 1 | | 0.51 |
|  | AATAT/ATATT | 2 | 1.02 | AATAT/ATATT | 2 | | 1.02 |
|  | ***P. cernua* f. *plumbea*** | | | ***P. dahurica*** | | | |
|  | **SSR repeat units** | **SSR abundances** | **Percent abundance (%)** | **SSR repeat units** | | **SSR abundances** | **Percent abundance (%)** |
| Mononucleotide | A/T | 157 | 75.85 | A/T | | 156 | 75.00 |
|  | C/G | 8 | 3.86 | C/G | | 8 | 3.85 |
| Dinucleotide | AT/AT | 14 | 6.76 | AT/AT | | 14 | 6.73 |
| Trinucleotide | AAT/ATT | 8 | 3.86 | AAT/ATT | | 8 | 3.85 |
|  | ACT/AGT | 2 | 0.97 | ACT/AGT | | 2 | 0.96 |
| Tetranucleotide | AAAG/CTTT | 2 | 0.97 | AAAG/CTTT | | 2 | 0.96 |
|  | AAAT/ATTT | 1 | 0.48 | AAAT/ATTT | | 1 | 0.48 |
|  | AACG/CGTT | 1 | 0.48 | AACG/CGTT | | 1 | 0.48 |
|  | AACT/AGTT | 4 | 1.93 | AACT/AGTT | | 4 | 1.92 |
|  | AAGT/ACTT | 1 | 0.48 | AAGT/ACTT | | 1 | 0.48 |
|  | AATG/ATTC | 1 | 0.48 | AATG/ATTC | | 1 | 0.48 |
|  | AATT/AATT | 3 | 1.45 | AATT/AATT | | 3 | 1.44 |
|  | AGAT/ATCT | 3 | 1.45 | AGAT/ATCT | | 3 | 1.44 |
| Pentanucleotide | AAAAT/ATTTT | 1 | 0.48 | AAAAT/ATTTT | | 1 | 0.48 |
|  | AATAT/ATATT | 1 | 0.48 | AATAT/ATATT | | 2 | 0.96 |
| Hexalnucleotide | - | - | - | AATATT/AATATT | | 1 | 0.48 |
|  | ***P. turczaninovii*** | | | ***P. cernua*** | | | |
|  | **SSR repeat units** | **SSR**  **abundances** | **Percent abundance (%)** | **SSR repeat units** | | **SSR abundances** | **Percent abundance (%)** |
| Mononucleotide | A/T | 150 | 76.53 | A/T | | 147 | 75.00 |
|  | C/G | 7 | 3.57 | C/G | | 8 | 4.08 |
| Dinucleotide | AT/AT | 8 | 4.08 | AT/AT | | 7 | 3.57 |
| Trinucleotide | AAG/CTT | 1 | 0.51 | AAT/ATT | | 12 | 6.12 |
|  | AAT/ATT | 8 | 4.08 | ACT/AGT | | 2 | 1.02 |
|  | ACT/AGT | 2 | 1.02 | - | | - | - |
| Tetranucleotide | AAAG/CTTT | 2 | 1.02 | AAAG/CTTT | | 2 | 1.02 |
|  | AAAT/ATTT | 3 | 1.53 | AAAT/ATTT | | 2 | 1.02 |
|  | AACG/CGTT | 1 | 0.51 | AACG/CGTT | | 1 | 0.51 |
|  | AACT/AGTT | 4 | 2.04 | AACT/AGTT | | 4 | 2.04 |
|  | AAGT/ACTT | 1 | 0.51 | AAGT/ACTT | | 1 | 0.51 |
|  | AATG/ATTC | 1 | 0.51 | AATG/ATTC | | 1 | 0.51 |
|  | AATT/AATT | 3 | 1.53 | AATT/AATT | | 3 | 1.53 |
|  | AGAT/ATCT | 2 | 1.02 | AGAT/ATCT | | 3 | 1.53 |
| Pentanucleotide | AAATT/AATTT | 1 | 0.51 | AATAC/ATTGT | | 1 | 0.51 |
|  | AATAT/ATATT | 2 | 1.02 | AATGT/ACATT | | 2 | 1.02 |
