# Supplementary material for: Comparative analysis of the complete chloroplast genome sequences of six species of Pulsatilla Miller, Ranunculaceae
Source: Chin Med. 2019 Nov 28;14:53. doi: 10.1186/s13020-019-0274-5 (PMC6883693; doi:10.1186/s13020-019-0274-5)
Supplement: Supplementary file 8 — Additional file 8: Table S3. SSRs distribution of the P. chinensis cp genome. [file 13020_2019_274_MOESM8_ESM.docx]

**Table S3 SSRs distribution of the *P. chinensis* cp genome**

| **SSR nr.** | **SSR Type** | **SSR** | **Size** | **Star** | **End** | **Location** |
| --- | --- | --- | --- | --- | --- | --- |
| 2 | p1 | (T)10 | 10 | 1600 | 1609 | CNS |
| 3 | p1 | (A)15 | 15 | 1984 | 1998 | CNS |
| 4 | p1 | (T)8 | 8 | 2619 | 2626 | CNS |
| 5 | p1 | (T)10 | 10 | 2820 | 2829 | CNS |
| 6 | p1 | (A)10 | 10 | 3073 | 3082 | CNS |
| 7 | p1 | (A)9 | 9 | 3592 | 3600 | CNS |
| 8 | p1 | (T)10 | 10 | 3855 | 3864 | CNS |
| 9 | p1 | (T)10 | 10 | 4425 | 4434 | CNS |
| 10 | p1 | (A)9 | 9 | 5452 | 5460 | *matK* |
| 11 | p1 | (T)8 | 8 | 5795 | 5802 | *matK* |
| 12 | p1 | (T)10 | 10 | 6115 | 6124 | *matK* |
| 16 | p2 | (AT)5 | 10 | 9481 | 9490 | CNS |
| 17 | p1 | (A)8 | 8 | 11275 | 11282 | CNS |
| 18 | p1 | (A)8 | 8 | 12304 | 12311 | *ycf3*-CDS2 |
| 19 | p5 | (TATTA)3 | 15 | 18059 | 18073 | CNS |
| 22 | p2 | (AT)6 | 12 | 19111 | 19122 | CNS |
| 23 | p1 | (C)10 | 10 | 20350 | 20359 | CNS |
| 24 | p5 | (TTTAC)3 | 15 | 22233 | 22247 | *psbC* |
| 25 | p1 | (A)8 | 8 | 22853 | 22860 | CNS |
| 26 | p1 | (A)10 | 10 | 23302 | 23311 | CNS |
| 27 | p1 | (T)9 | 9 | 23559 | 23567 | CNS |
| 28 | p1 | (A)9 | 9 | 23757 | 23765 | CNS |
| 29 | p3 | (ATA)4 | 12 | 23891 | 23902 | CNS |
| 31 | p1 | (T)15 | 15 | 24851 | 24865 | CNS |
| 32 | p2 | (AT)5 | 10 | 25545 | 25554 | CNS |
| 34 | p1 | (A)8 | 8 | 28421 | 28428 | CNS |
| 35 | p1 | (A)10 | 10 | 30132 | 30141 | CNS |
| 36 | p1 | (T)8 | 8 | 33425 | 33432 | *rpoB* |
| 37 | p1 | (A)8 | 8 | 33798 | 33805 | CNS |
| 38 | p1 | (T)8 | 8 | 34019 | 34026 | CNS |
| 39 | p1 | (A)9 | 9 | 35186 | 35194 | *rpoC1*-CDS2 |
| 40 | p2 | (AT)5 | 10 | 36458 | 36467 | *rpoC1*-CDS2 |
| 41 | p1 | (G)8 | 8 | 37305 | 37312 | *rpoC2* |
| 42 | p1 | (T)8 | 8 | 37690 | 37697 | *rpoC2* |
| 43 | p1 | (A)14 | 14 | 37828 | 37841 | *rpoC2* |
| 44 | p1 | (A)9 | 9 | 38004 | 38012 | *rpoC2* |
| 45 | p1 | (A)8 | 8 | 38228 | 38235 | *rpoC2* |
| 46 | p1 | (T)8 | 8 | 38661 | 38668 | *rpoC2* |
| 47 | p1 | (A)8 | 8 | 40024 | 40031 | *rpoC2* |
| 49 | p3 | (TTA)4 | 12 | 43165 | 43176 | *rps2* |
| 51 | p1 | (T)8 | 8 | 43736 | 43743 | *atpF*-CDS1 |
| 52 | p1 | (A)9 | 9 | 44198 | 44206 | CNS |
| 53 | p1 | (T)10 | 10 | 46630 | 46639 | *atpA* |
| 54 | p1 | (A)9 | 9 | 47369 | 47377 | CNS |
| 55 | p1 | (T)9 | 9 | 47506 | 47514 | CNS |
| 56 | p1 | (A)8 | 8 | 47769 | 47776 | CNS |
| 57 | p1 | (A)8 | 8 | 48257 | 48264 | CNS |
| 58 | p1 | (A)8 | 8 | 49140 | 49147 | CNS |
| 60 | p1 | (T)9 | 9 | 51375 | 51383 | *ndhJ* |
| 61 | p1 | (A)10 | 10 | 52900 | 52909 | CNS |
| 63 | p1 | (T)14 | 14 | 54534 | 54547 | CNS |
| 64 | p1 | (T)9 | 9 | 56913 | 56921 | *atpB* |
| 65 | p1 | (A)9 | 9 | 57287 | 57295 | CNS |
| 66 | p1 | (T)8 | 8 | 59414 | 59421 | CNS |
| 67 | p1 | (T)8 | 8 | 60227 | 60234 | *accD* |
| 69 | p1 | (A)10 | 10 | 61754 | 61763 | CNS |
| 70 | p1 | (T)13 | 13 | 62158 | 62170 | *psaI* |
| 71 | p1 | (A)8 | 8 | 62550 | 62557 | *ycf4* |
| 72 | p1 | (T)9 | 9 | 63460 | 63468 | CNS |
| 73 | p1 | (T)9 | 9 | 64598 | 64606 | *cemA* |
| 74 | p1 | (A)8 | 8 | 65165 | 65172 | *petA* |
| 75 | p1 | (A)8 | 8 | 65781 | 65788 | *petA* |
| 76 | p1 | (A)8 | 8 | 67833 | 67840 | CNS |
| 77 | p1 | (T)11 | 11 | 67974 | 67984 | CNS |
| 79 | p1 | (A)11 | 11 | 69447 | 69457 | *psaJ* |
| 80 | p1 | (A)9 | 9 | 70029 | 70037 | *rpl33* |
| 84 | p1 | (T)14 | 14 | 72334 | 72347 | *rps12*-D2-CDS1; *clpP*-CDS1 |
| 86 | p1 | (A)10 | 10 | 77658 | 77667 | CNS |
| 87 | p1 | (A)8 | 8 | 77995 | 78002 | CNS |
| 88 | p1 | (T)11 | 11 | 79759 | 79769 | CNS |
| 89 | p1 | (A)8 | 8 | 80591 | 80598 | *rpoA* |
| 90 | p1 | (T)10 | 10 | 80820 | 80829 | *rpoA* |
| 91 | p1 | (A)8 | 8 | 81527 | 81534 | *rpoA* |
| 93 | p1 | (T)9 | 9 | 83100 | 83108 | *rps8* |
| 94 | p4 | (CTAA)3 | 12 | 83666 | 83677 | *rpl14*; *rpl16*-CDS1 |
| 97 | p1 | (G)10 | 10 | 87689 | 87698 | CNS |
| 98 | p1 | (A)9 | 9 | 92171 | 92179 | *ycf2* |
| 100 | p1 | (A)8 | 8 | 102787 | 102794 | CNS |
| 101 | p1 | (C)9 | 9 | 103004 | 103012 | CNS |
| 102 | p1 | (A)9 | 9 | 110513 | 110521 | CNS |
| 103 | p1 | (T)8 | 8 | 110908 | 110915 | CNS |
| 104 | p1 | (T)8 | 8 | 112991 | 112998 | CNS |
| 105 | p5 | (AAATA)3 | 15 | 113526 | 113540 | CNS |
| 106 | p4 | (TAAA)3 | 12 | 113761 | 113772 | CNS |
| 109 | p5 | (AATAT)3 | 15 | 116763 | 116777 | CNS |
| 110 | p1 | (A)16 | 16 | 116913 | 116928 | CNS |
| 111 | p1 | (A)9 | 9 | 117151 | 117159 | CNS |
| 113 | p1 | (A)10 | 10 | 117709 | 117718 | CNS |
| 114 | p4 | (TAAG)3 | 12 | 118501 | 118512 | CNS |
| 116 | p1 | (A)8 | 8 | 120623 | 120630 | *ndhD* |
| 117 | p1 | (A)8 | 8 | 121415 | 121422 | *ndhD* |
| 118 | p1 | (T)8 | 8 | 122216 | 122223 | *psaC* |
| 120 | p1 | (A)9 | 9 | 123204 | 123212 | *ndhG* |
| 121 | p1 | (A)8 | 8 | 123596 | 123603 | *ndhG* |
| 122 | p1 | (T)10 | 10 | 123780 | 123789 | CNS |
| 123 | p1 | (A)8 | 8 | 125850 | 125857 | CNS |
| 125 | p1 | (A)8 | 8 | 127997 | 128004 | *rps15* |
| 126 | p1 | (T)10 | 10 | 129091 | 129100 | *ycf1* |
| 127 | p1 | (T)13 | 13 | 129535 | 129547 | *ycf1* |
| 128 | p1 | (T)9 | 9 | 129651 | 129659 | *ycf1* |
| 129 | p4 | (CATT)3 | 12 | 130543 | 130554 | *ycf1* |
| 130 | p1 | (T)10 | 10 | 131122 | 131131 | *ycf1* |
| 131 | p1 | (T)16 | 16 | 131280 | 131295 | *ycf1* |
| 134 | p1 | (T)9 | 9 | 132460 | 132468 | *ycf1* |
| 135 | p1 | (A)9 | 9 | 132577 | 132585 | *ycf1* |
| 136 | p1 | (A)8 | 8 | 133196 | 133203 | *ycf1* |
| 137 | p1 | (A)8 | 8 | 135279 | 135286 | CNS |
| 138 | p1 | (T)9 | 9 | 135673 | 135681 | CNS |
| 139 | p1 | (G)9 | 9 | 143182 | 143190 | CNS |
| 140 | p1 | (T)8 | 8 | 143400 | 143407 | CNS |
| 142 | p1 | (T)9 | 9 | 154015 | 154023 | *ycf2*-D2 |
| 143 | p1 | (C)10 | 10 | 158496 | 158505 | CNS |
| 146 | p4 | (TTAG)3 | 12 | 162517 | 162528 | *rpl14*-D2; *rpl16*-D2-CDS2 |
| 147 | p1 | (A)9 | 9 | 163086 | 163094 | *rps8*-D2 |

**SSR simple sequence repeats, CDS coding sequences, CNS non-coding sequences**
