# Supplementary material for: Comparative analysis of the complete chloroplast genome sequences of six species of Pulsatilla Miller, Ranunculaceae
Source: Chin Med. 2019 Nov 28;14:53. doi: 10.1186/s13020-019-0274-5 (PMC6883693; doi:10.1186/s13020-019-0274-5)
Supplement: Supplementary file 9 — Additional file 9: Table S4. SSRs distribution of the P. chinensis var. kissii cp genome. [file 13020_2019_274_MOESM9_ESM.docx]

**Table S4 SSRs distribution of the *P. chinensis* var. *kissii* cp genome**

| **SSR nr.** | **SSR Type** | **SSR** | **Size** | **Star** | **End** | **Location** |
| --- | --- | --- | --- | --- | --- | --- |
| 2 | p1 | (T)10 | 10 | 1600 | 1609 | CNS |
| 3 | p1 | (A)15 | 15 | 1984 | 1998 | CNS |
| 4 | p1 | (T)8 | 8 | 2619 | 2626 | CNS |
| 5 | p1 | (T)11 | 11 | 2820 | 2830 | CNS |
| 6 | p1 | (A)10 | 10 | 3078 | 3087 | CNS |
| 7 | p1 | (A)9 | 9 | 3597 | 3605 | CNS |
| 8 | p1 | (T)10 | 10 | 3860 | 3869 | CNS |
| 9 | p1 | (T)10 | 10 | 4430 | 4439 | *matK* |
| 10 | p1 | (A)9 | 9 | 5457 | 5465 | *matK* |
| 11 | p1 | (T)8 | 8 | 5800 | 5807 | *matK* |
| 12 | p1 | (T)10 | 10 | 6120 | 6129 | CNS |
| 16 | p2 | (AT)5 | 10 | 9486 | 9495 | CNS |
| 17 | p1 | (A)8 | 8 | 11280 | 11287 | *ycf3*-CDS2 |
| 18 | p1 | (A)8 | 8 | 12309 | 12316 | CNS |
| 19 | p5 | (TATTA)3 | 15 | 18064 | 18078 | CNS |
| 22 | p2 | (AT)6 | 12 | 19116 | 19127 | CNS |
| 23 | p1 | (C)10 | 10 | 20355 | 20364 | *psbC* |
| 24 | p5 | (TTTAC)3 | 15 | 22238 | 22252 | CNS |
| 25 | p1 | (A)8 | 8 | 22858 | 22865 | CNS |
| 26 | p1 | (A)10 | 10 | 23307 | 23316 | CNS |
| 27 | p1 | (T)9 | 9 | 23564 | 23572 | CNS |
| 28 | p1 | (A)9 | 9 | 23762 | 23770 | CNS |
| 29 | p3 | (ATA)4 | 12 | 23896 | 23907 | CNS |
| 31 | p1 | (T)11 | 11 | 24810 | 24820 | CNS |
| 32 | p2 | (AT)5 | 10 | 25500 | 25509 | CNS |
| 34 | p1 | (A)8 | 8 | 28376 | 28383 | CNS |
| 35 | p1 | (A)10 | 10 | 30087 | 30096 | *rpoB* |
| 36 | p1 | (T)8 | 8 | 33380 | 33387 | CNS |
| 37 | p1 | (A)8 | 8 | 33753 | 33760 | CNS |
| 38 | p1 | (T)8 | 8 | 33974 | 33981 | *rpoC1*-CDS2 |
| 39 | p1 | (A)9 | 9 | 35141 | 35149 | *rpoC1*-CDS2 |
| 40 | p2 | (AT)5 | 10 | 36413 | 36422 | *rpoC2* |
| 41 | p1 | (G)8 | 8 | 37260 | 37267 | *rpoC2* |
| 42 | p1 | (T)8 | 8 | 37645 | 37652 | *rpoC2* |
| 43 | p1 | (A)14 | 14 | 37783 | 37796 | *rpoC2* |
| 44 | p1 | (A)9 | 9 | 37959 | 37967 | *rpoC2* |
| 45 | p1 | (A)8 | 8 | 38183 | 38190 | *rpoC2* |
| 46 | p1 | (T)8 | 8 | 38616 | 38623 | *rpoC2* |
| 47 | p1 | (A)8 | 8 | 39979 | 39986 | *rps2* |
| 49 | p3 | (TTA)4 | 12 | 43119 | 43130 | *atpH* |
| 51 | p1 | (T)8 | 8 | 43675 | 43682 | *atpF*-CDS1 |
| 52 | p1 | (A)9 | 9 | 44137 | 44145 | CNS |
| 53 | p1 | (T)10 | 10 | 46563 | 46572 | *atpA* |
| 54 | p1 | (A)9 | 9 | 47302 | 47310 | CNS |
| 55 | p1 | (T)9 | 9 | 47439 | 47447 | CNS |
| 56 | p1 | (A)8 | 8 | 47702 | 47709 | CNS |
| 57 | p1 | (A)8 | 8 | 48190 | 48197 | CNS |
| 58 | p1 | (A)8 | 8 | 49073 | 49080 | CNS |
| 60 | p1 | (T)9 | 9 | 51308 | 51316 | *ndhJ* |
| 61 | p1 | (A)10 | 10 | 52833 | 52842 | CNS |
| 63 | p1 | (T)14 | 14 | 54473 | 54486 | CNS |
| 64 | p1 | (T)9 | 9 | 56852 | 56860 | *atpB* |
| 65 | p1 | (A)9 | 9 | 57226 | 57234 | CNS |
| 66 | p1 | (T)8 | 8 | 59353 | 59360 | CNS |
| 67 | p1 | (T)8 | 8 | 60166 | 60173 | *accD* |
| 69 | p1 | (A)10 | 10 | 61693 | 61702 | CNS |
| 70 | p1 | (T)13 | 13 | 62097 | 62109 | *psaI* |
| 71 | p1 | (A)8 | 8 | 62489 | 62496 | *ycf4* |
| 72 | p1 | (T)9 | 9 | 63399 | 63407 | CNS |
| 73 | p1 | (T)9 | 9 | 64537 | 64545 | *cemA* |
| 74 | p1 | (A)8 | 8 | 65104 | 65111 | *petA* |
| 75 | p1 | (A)8 | 8 | 65720 | 65727 | *petA* |
| 76 | p1 | (A)8 | 8 | 67777 | 67784 | CNS |
| 77 | p1 | (T)9 | 9 | 67918 | 67926 | CNS |
| 79 | p1 | (A)11 | 11 | 69398 | 69408 | *psaJ* |
| 80 | p1 | (A)9 | 9 | 69980 | 69988 | *rpl33* |
| 84 | p1 | (T)15 | 15 | 72285 | 72299 | *rps12*-D2-CDS1; *clpP*-CDS1 |
| 86 | p1 | (A)10 | 10 | 77610 | 77619 | CNS |
| 87 | p1 | (A)8 | 8 | 77947 | 77954 | CNS |
| 88 | p1 | (T)11 | 11 | 79711 | 79721 | CNS |
| 89 | p1 | (A)8 | 8 | 80543 | 80550 | *rpoA* |
| 90 | p1 | (T)10 | 10 | 80772 | 80781 | *rpoA* |
| 91 | p1 | (A)8 | 8 | 81479 | 81486 | *rpoA* |
| 93 | p1 | (T)9 | 9 | 83052 | 83060 | *rps8* |
| 94 | p4 | (CTAA)3 | 12 | 83618 | 83629 | *rpl16*-CDS1; *rpl14* |
| 97 | p1 | (G)10 | 10 | 87641 | 87650 | CNS |
| 98 | p1 | (A)9 | 9 | 92123 | 92131 | *ycf2* |
| 100 | p1 | (A)8 | 8 | 102739 | 102746 | CNS |
| 101 | p1 | (C)9 | 9 | 102956 | 102964 | CNS |
| 102 | p1 | (A)9 | 9 | 110465 | 110473 | CNS |
| 103 | p1 | (T)8 | 8 | 110860 | 110867 | CNS |
| 104 | p1 | (T)8 | 8 | 112943 | 112950 | CNS |
| 105 | p5 | (AAATA)3 | 15 | 113478 | 113492 | CNS |
| 106 | p4 | (TAAA)3 | 12 | 113713 | 113724 | CNS |
| 109 | p5 | (AATAT)3 | 15 | 116652 | 116666 | CNS |
| 110 | p1 | (A)16 | 16 | 116802 | 116817 | CNS |
| 111 | p1 | (A)9 | 9 | 117040 | 117048 | CNS |
| 113 | p1 | (A)9 | 9 | 117606 | 117614 | CNS |
| 114 | p4 | (TAAG)3 | 12 | 118397 | 118408 | CNS |
| 115 | p1 | (T)8 | 8 | 119273 | 119280 | *ccsA* |
| 117 | p1 | (A)8 | 8 | 120519 | 120526 | *ndhD* |
| 118 | p1 | (A)8 | 8 | 121311 | 121318 | *ndhD* |
| 119 | p1 | (T)8 | 8 | 122112 | 122119 | *psaC* |
| 121 | p1 | (A)9 | 9 | 123100 | 123108 | *ndhG* |
| 122 | p1 | (A)8 | 8 | 123492 | 123499 | *ndhG* |
| 123 | p1 | (T)10 | 10 | 123676 | 123685 | CNS |
| 124 | p1 | (A)8 | 8 | 125746 | 125753 | CNS |
| 126 | p1 | (A)8 | 8 | 127893 | 127900 | *rps15* |
| 127 | p1 | (T)10 | 10 | 128987 | 128996 | *ycf1* |
| 128 | p1 | (T)13 | 13 | 129431 | 129443 | *ycf1* |
| 129 | p1 | (T)9 | 9 | 129547 | 129555 | *ycf1* |
| 130 | p4 | (CATT)3 | 12 | 130439 | 130450 | *ycf1* |
| 131 | p1 | (T)10 | 10 | 131018 | 131027 | *ycf1* |
| 132 | p1 | (T)16 | 16 | 131176 | 131191 | *ycf1* |
| 135 | p1 | (T)9 | 9 | 132356 | 132364 | *ycf1* |
| 136 | p1 | (A)9 | 9 | 132473 | 132481 | *ycf1* |
| 137 | p1 | (A)8 | 8 | 133101 | 133108 | *ycf1* |
| 138 | p1 | (A)8 | 8 | 135184 | 135191 | CNS |
| 139 | p1 | (T)9 | 9 | 135578 | 135586 | CNS |
| 140 | p1 | (G)9 | 9 | 143087 | 143095 | CNS |
| 141 | p1 | (T)8 | 8 | 143305 | 143312 | CNS |
| 143 | p1 | (T)9 | 9 | 153920 | 153928 | *ycf2*-D2 |
| 144 | p1 | (C)10 | 10 | 158401 | 158410 | CNS |
| 147 | p4 | (TTAG)3 | 12 | 162422 | 162433 | *rpl14*-D2; *rpl16*-D2-CDS2 |
| 148 | p1 | (A)9 | 9 | 162991 | 162999 | *rps8*-D2 |

**SSR simple sequence repeats, CDS coding sequences, CNS non-coding sequences**
