# Supplementary material for: Comparative analysis of the complete chloroplast genome sequences of six species of Pulsatilla Miller, Ranunculaceae
Source: Chin Med. 2019 Nov 28;14:53. doi: 10.1186/s13020-019-0274-5 (PMC6883693; doi:10.1186/s13020-019-0274-5)
Supplement: Supplementary file 10 — Additional file 10: Table S5. SSRs distribution of the P. cernua f. plumbea cp genome. [file 13020_2019_274_MOESM10_ESM.docx]

**Table S5 SSRs distribution of the *P. cernua* f. *plumbea* cp genome**

| **SSR nr.** | **SSR Type** | **SSR** | **Size** | **Star** | **End** | **Location** |
| --- | --- | --- | --- | --- | --- | --- |
| 3 | p2 | (TA)5 | 10 | 1523 | 1532 | *rps16*-CDS1 |
| 4 | p1 | (T)12 | 12 | 1795 | 1806 | CNS |
| 5 | p1 | (A)14 | 14 | 2181 | 2194 | CNS |
| 6 | p1 | (T)9 | 9 | 3016 | 3024 | CNS |
| 7 | p1 | (A)12 | 12 | 3129 | 3140 | CNS |
| 8 | p1 | (A)10 | 10 | 3693 | 3702 | CNS |
| 9 | p1 | (T)10 | 10 | 3950 | 3959 | CNS |
| 10 | p4 | (AGAT)3 | 12 | 4298 | 4309 | *matK* |
| 11 | p1 | (T)10 | 10 | 4520 | 4529 | *matK* |
| 12 | p1 | (T)9 | 9 | 4874 | 4882 | *matK* |
| 13 | p1 | (A)9 | 9 | 5541 | 5549 | *matK* |
| 14 | p1 | (T)9 | 9 | 6157 | 6165 | CNS |
| 20 | p1 | (A)8 | 8 | 12337 | 12344 | CNS |
| 24 | p2 | (AT)6 | 12 | 19124 | 19135 | CNS |
| 25 | p1 | (C)10 | 10 | 20363 | 20372 | *psbC* |
| 26 | p1 | (A)8 | 8 | 22080 | 22087 | CNS |
| 27 | p4 | (ATCT)3 | 12 | 22534 | 22545 | CNS |
| 28 | p1 | (A)8 | 8 | 22791 | 22798 | CNS |
| 29 | p1 | (A)11 | 11 | 23204 | 23214 | CNS |
| 30 | p1 | (T)9 | 9 | 23462 | 23470 | CNS |
| 31 | p3 | (ATA)4 | 12 | 23792 | 23803 | CNS |
| 32 | p1 | (T)9 | 9 | 24365 | 24373 | CNS |
| 34 | p2 | (AT)5 | 10 | 25287 | 25296 | CNS |
| 36 | p1 | (A)8 | 8 | 28130 | 28137 | CNS |
| 37 | p1 | (A)10 | 10 | 29841 | 29850 | *rpoB* |
| 38 | p1 | (T)8 | 8 | 33134 | 33141 | CNS |
| 39 | p1 | (A)8 | 8 | 33507 | 33514 | CNS |
| 40 | p1 | (T)8 | 8 | 33728 | 33735 | *rpo*C1-CDS2 |
| 41 | p1 | (A)9 | 9 | 34895 | 34903 | *rpo*C1-CDS2 |
| 42 | p2 | (AT)5 | 10 | 36166 | 36175 | *rpoC2* |
| 43 | p1 | (G)8 | 8 | 37013 | 37020 | *rpoC2* |
| 44 | p1 | (T)8 | 8 | 37398 | 37405 | *rpoC2* |
| 45 | p1 | (A)14 | 14 | 37536 | 37549 | *rpoC2* |
| 46 | p1 | (A)9 | 9 | 37712 | 37720 | *rpoC2* |
| 47 | p1 | (A)8 | 8 | 37936 | 37943 | *rpoC2* |
| 48 | p1 | (T)8 | 8 | 38369 | 38376 | *rpoC2* |
| 49 | p1 | (A)8 | 8 | 39732 | 39739 | *rps2* |
| 52 | p1 | (T)8 | 8 | 43431 | 43438 | *atpF*-CDS1 |
| 53 | p1 | (A)10 | 10 | 43893 | 43902 | CNS |
| 54 | p1 | (T)8 | 8 | 46319 | 46326 | *atpA* |
| 56 | p1 | (A)13 | 13 | 47463 | 47475 | CNS |
| 57 | p1 | (A)8 | 8 | 47946 | 47953 | CNS |
| 60 | p1 | (A)8 | 8 | 50374 | 50381 | CNS |
| 61 | p1 | (T)9 | 9 | 51103 | 51111 | *ndhJ* |
| 62 | p1 | (A)11 | 11 | 52637 | 52647 | CNS |
| 64 | p1 | (T)10 | 10 | 54344 | 54353 | CNS |
| 65 | p1 | (T)11 | 11 | 54542 | 54552 | CNS |
| 66 | p1 | (T)10 | 10 | 56669 | 56678 | *atpB* |
| 67 | p1 | (A)9 | 9 | 57045 | 57053 | CNS |
| 68 | p1 | (T)8 | 8 | 59151 | 59158 | CNS |
| 69 | p1 | (T)8 | 8 | 59964 | 59971 | *accD* |
| 70 | p1 | (T)8 | 8 | 61099 | 61106 | *accD* |
| 71 | p1 | (A)8 | 8 | 61468 | 61475 | CNS |
| 72 | p1 | (T)16 | 16 | 61889 | 61904 | *psaI* |
| 73 | p1 | (A)8 | 8 | 62274 | 62281 | *ycf4* |
| 74 | p1 | (A)8 | 8 | 63357 | 63364 | CNS |
| 75 | p1 | (T)10 | 10 | 64302 | 64311 | *cemA* |
| 76 | p1 | (A)8 | 8 | 64870 | 64877 | *petA* |
| 77 | p1 | (A)9 | 9 | 65486 | 65494 | *petA* |
| 78 | p5 | (ATTAT)3 | 15 | 67027 | 67041 | CNS |
| 79 | p1 | (A)8 | 8 | 67462 | 67469 | CNS |
| 81 | p1 | (A)13 | 13 | 69093 | 69105 | *psaJ* |
| 82 | p1 | (A)9 | 9 | 69698 | 69706 | *rpl33* |
| 83 | p1 | (A)9 | 9 | 70051 | 70059 | *rps18* |
| 86 | p1 | (T)16 | 16 | 72012 | 72027 | *rps12*-D2-CDS1; *clpP*-CDS1 |
| 88 | p1 | (A)16 | 16 | 77284 | 77299 | CNS |
| 89 | p1 | (A)14 | 14 | 77578 | 77591 | CNS |
| 90 | p1 | (T)16 | 16 | 79348 | 79363 | CNS |
| 91 | p1 | (A)8 | 8 | 80177 | 80184 | *rpoA* |
| 92 | p1 | (T)10 | 10 | 80406 | 80415 | *rpoA* |
| 93 | p1 | (A)8 | 8 | 81113 | 81120 | *rpoA* |
| 95 | p1 | (T)9 | 9 | 82682 | 82690 | *rps8* |
| 96 | p4 | (CTAA)3 | 12 | 83248 | 83259 | *rpl16*-CDS1; *rpl14* |
| 99 | p1 | (G)12 | 12 | 87261 | 87272 | CNS |
| 100 | p1 | (A)9 | 9 | 91745 | 91753 | *ycf2* |
| 103 | p1 | (A)8 | 8 | 102424 | 102431 | CNS |
| 104 | p1 | (C)9 | 9 | 102641 | 102649 | CNS |
| 105 | p1 | (A)9 | 9 | 110150 | 110158 | CNS |
| 106 | p1 | (T)8 | 8 | 110541 | 110548 | CNS |
| 107 | p1 | (T)8 | 8 | 112624 | 112631 | CNS |
| 108 | p3 | (TAC)4 | 12 | 113095 | 113106 | CNS |
| 109 | p1 | (T)9 | 9 | 113248 | 113256 | CNS |
| 112 | p1 | (A)9 | 9 | 116062 | 116070 | CNS |
| 113 | p1 | (A)8 | 8 | 116281 | 116288 | CNS |
| 115 | p1 | (A)14 | 14 | 116887 | 116900 | CNS |
| 116 | p4 | (TAAG)3 | 12 | 117357 | 117368 | CNS |
| 117 | p1 | (T)8 | 8 | 118233 | 118240 | *ccsA* |
| 118 | p3 | (ATA)4 | 12 | 118882 | 118893 | CNS |
| 119 | p5 | (AATAA)3 | 15 | 119012 | 119026 | *ndhD* |
| 120 | p1 | (A)8 | 8 | 119364 | 119371 | *ndhD* |
| 121 | p1 | (A)8 | 8 | 120156 | 120163 | *ndhD* |
| 122 | p1 | (T)8 | 8 | 120957 | 120964 | *psaC* |
| 124 | p1 | (A)9 | 9 | 121937 | 121945 | *ndhG* |
| 125 | p1 | (A)8 | 8 | 122329 | 122336 | *ndhG* |
| 126 | p1 | (T)13 | 13 | 122513 | 122525 | CNS |
| 127 | p1 | (A)8 | 8 | 124609 | 124616 | *ndhA*-CDS2 |
| 128 | p2 | (TA)8 | 16 | 126537 | 126552 | *ndhH*; *rps15* |
| 129 | p1 | (T)13 | 13 | 128125 | 128137 | *ycf1* |
| 130 | p1 | (T)9 | 9 | 128241 | 128249 | *ycf1* |
| 131 | p4 | (CATT)3 | 12 | 129109 | 129120 | *ycf1* |
| 132 | p1 | (T)10 | 10 | 129694 | 129703 | *ycf1* |
| 133 | p1 | (T)16 | 16 | 129852 | 129867 | *ycf1* |
| 135 | p1 | (T)9 | 9 | 130554 | 130562 | *ycf1* |
| 136 | p1 | (A)8 | 8 | 130667 | 130674 | *ycf1* |
| 138 | p3 | (AGT)4 | 12 | 131298 | 131309 | *ycf1* |
| 139 | p1 | (A)8 | 8 | 131774 | 131781 | *ycf1* |
| 140 | p1 | (A)8 | 8 | 133857 | 133864 | CNS |
| 141 | p1 | (T)9 | 9 | 134247 | 134255 | CNS |
| 142 | p1 | (G)9 | 9 | 141756 | 141764 | CNS |
| 143 | p1 | (T)8 | 8 | 141974 | 141981 | CNS |
| 146 | p1 | (T)9 | 9 | 152652 | 152660 | *ycf2*-D2 |
| 147 | p1 | (C)12 | 12 | 157133 | 157144 | CNS |
| 150 | p4 | (TTAG)3 | 12 | 161146 | 161157 | *rpl14*-D2; *rpl16*-D2-CDS2 |
| 151 | p1 | (A)9 | 9 | 161715 | 161723 | *rps8*-D2 |

**SSR simple sequence repeats, CDS coding sequences, CNS non-coding sequences**
