# Supplementary material for: Comparative analysis of the complete chloroplast genome sequences of six species of Pulsatilla Miller, Ranunculaceae
Source: Chin Med. 2019 Nov 28;14:53. doi: 10.1186/s13020-019-0274-5 (PMC6883693; doi:10.1186/s13020-019-0274-5)
Supplement: Supplementary file 11 — Additional file 11: Table S6. SSRs distribution of the P. dahurica cp genome. [file 13020_2019_274_MOESM11_ESM.docx]

**Table S6 SSRs distribution of the *P. dahurica* cp genome**

| **SSR nr.** | **SSR Type** | **SSR** | **Size** | **Star** | **End** | **Location** |
| --- | --- | --- | --- | --- | --- | --- |
| 3 | p2 | (TA)5 | 10 | 1529 | 1538 | *rps16*-CDS1 |
| 4 | p1 | (T)13 | 13 | 1800 | 1812 | CNS |
| 5 | p1 | (A)13 | 13 | 2187 | 2199 | CNS |
| 6 | p1 | (T)9 | 9 | 3021 | 3029 | CNS |
| 7 | p1 | (A)12 | 12 | 3134 | 3145 | CNS |
| 8 | p1 | (A)9 | 9 | 3698 | 3706 | CNS |
| 9 | p1 | (T)10 | 10 | 3954 | 3963 | CNS |
| 10 | p4 | (AGAT)3 | 12 | 4302 | 4313 | *matK* |
| 11 | p1 | (T)10 | 10 | 4524 | 4533 | *matK* |
| 12 | p1 | (T)9 | 9 | 4878 | 4886 | *matK* |
| 13 | p1 | (A)9 | 9 | 5545 | 5553 | *matK* |
| 14 | p1 | (T)9 | 9 | 6161 | 6169 | CNS |
| 20 | p1 | (A)8 | 8 | 12341 | 12348 | CNS |
| 24 | p2 | (AT)6 | 12 | 19120 | 19131 | CNS |
| 25 | p1 | (C)10 | 10 | 20359 | 20368 | *psbC* |
| 26 | p1 | (A)8 | 8 | 22076 | 22083 | CNS |
| 27 | p4 | (ATCT)3 | 12 | 22530 | 22541 | CNS |
| 28 | p1 | (A)8 | 8 | 22787 | 22794 | CNS |
| 29 | p1 | (A)11 | 11 | 23200 | 23210 | CNS |
| 30 | p1 | (T)9 | 9 | 23458 | 23466 | CNS |
| 31 | p3 | (ATA)4 | 12 | 23788 | 23799 | CNS |
| 32 | p1 | (T)9 | 9 | 24361 | 24369 | CNS |
| 34 | p2 | (AT)5 | 10 | 25272 | 25281 | CNS |
| 36 | p1 | (A)8 | 8 | 28115 | 28122 | CNS |
| 37 | p1 | (A)10 | 10 | 29826 | 29835 | *rpoB* |
| 38 | p1 | (T)8 | 8 | 33119 | 33126 | CNS |
| 39 | p1 | (A)8 | 8 | 33492 | 33499 | CNS |
| 40 | p1 | (T)8 | 8 | 33713 | 33720 | *rpoC1*-CDS2 |
| 41 | p1 | (A)9 | 9 | 34880 | 34888 | *rpoC1*-CDS2 |
| 42 | p2 | (AT)5 | 10 | 36151 | 36160 | *rpoC2* |
| 43 | p1 | (G)8 | 8 | 36998 | 37005 | *rpoC2* |
| 44 | p1 | (T)8 | 8 | 37383 | 37390 | *rpoC2* |
| 45 | p1 | (A)14 | 14 | 37521 | 37534 | *rpoC2* |
| 46 | p1 | (A)9 | 9 | 37697 | 37705 | *rpoC2* |
| 47 | p1 | (A)8 | 8 | 37921 | 37928 | *rpoC2* |
| 48 | p1 | (T)8 | 8 | 38354 | 38361 | *rpoC2* |
| 49 | p1 | (A)8 | 8 | 39717 | 39724 | *rps2* |
| 52 | p1 | (T)8 | 8 | 43416 | 43423 | *atpF*-CDS1 |
| 53 | p1 | (A)10 | 10 | 43878 | 43887 | CNS |
| 54 | p1 | (T)8 | 8 | 46304 | 46311 | *atpA* |
| 56 | p1 | (A)13 | 13 | 47448 | 47460 | CNS |
| 57 | p1 | (A)8 | 8 | 47931 | 47938 | CNS |
| 60 | p1 | (A)8 | 8 | 50361 | 50368 | CNS |
| 61 | p1 | (T)9 | 9 | 51090 | 51098 | *ndhJ* |
| 64 | p1 | (T)10 | 10 | 54327 | 54336 | CNS |
| 65 | p1 | (T)11 | 11 | 54525 | 54535 | CNS |
| 66 | p1 | (T)10 | 10 | 56652 | 56661 | *atpB* |
| 67 | p1 | (A)9 | 9 | 57028 | 57036 | CNS |
| 68 | p1 | (T)8 | 8 | 59134 | 59141 | CNS |
| 69 | p1 | (T)8 | 8 | 59947 | 59954 | *accD* |
| 70 | p1 | (T)8 | 8 | 61082 | 61089 | *accD* |
| 71 | p1 | (A)8 | 8 | 61451 | 61458 | CNS |
| 72 | p1 | (T)16 | 16 | 61852 | 61867 | *psaI* |
| 73 | p1 | (A)8 | 8 | 62237 | 62244 | *ycf4* |
| 74 | p1 | (A)8 | 8 | 63320 | 63327 | CNS |
| 75 | p1 | (T)10 | 10 | 64265 | 64274 | *cemA* |
| 76 | p1 | (A)8 | 8 | 64833 | 64840 | *petA* |
| 78 | p5 | (ATTAT)3 | 15 | 66995 | 67009 | CNS |
| 79 | p1 | (A)8 | 8 | 67430 | 67437 | CNS |
| 81 | p1 | (A)13 | 13 | 69061 | 69073 | *psaJ* |
| 82 | p1 | (A)9 | 9 | 69666 | 69674 | *rpl33* |
| 83 | p1 | (A)9 | 9 | 70019 | 70027 | *rps18* |
| 86 | p1 | (T)16 | 16 | 71984 | 71999 | *clpP*-CDS1; *rps12*-D2-CDS1 |
| 88 | p1 | (A)16 | 16 | 77256 | 77271 | CNS |
| 89 | p1 | (A)14 | 14 | 77550 | 77563 | CNS |
| 90 | p1 | (T)15 | 15 | 79320 | 79334 | CNS |
| 91 | p1 | (A)8 | 8 | 80148 | 80155 | *rpoA* |
| 92 | p1 | (T)10 | 10 | 80377 | 80386 | *rpoA* |
| 93 | p1 | (A)8 | 8 | 81084 | 81091 | *rpoA* |
| 95 | p1 | (T)9 | 9 | 82653 | 82661 | *rps8* |
| 96 | p4 | (CTAA)3 | 12 | 83219 | 83230 | *rpl14*; *rpl16*-CDS1 |
| 99 | p1 | (G)12 | 12 | 87232 | 87243 | CNS |
| 100 | p1 | (A)9 | 9 | 91716 | 91724 | *ycf2* |
| 103 | p1 | (A)8 | 8 | 102395 | 102402 | CNS |
| 104 | p1 | (C)9 | 9 | 102612 | 102620 | CNS |
| 105 | p1 | (A)9 | 9 | 110121 | 110129 | CNS |
| 106 | p1 | (T)8 | 8 | 110512 | 110519 | CNS |
| 107 | p1 | (T)8 | 8 | 112595 | 112602 | CNS |
| 108 | p3 | (TAC)4 | 12 | 113066 | 113077 | CNS |
| 109 | p1 | (T)9 | 9 | 113219 | 113227 | CNS |
| 112 | p1 | (A)9 | 9 | 116017 | 116025 | CNS |
| 113 | p1 | (A)8 | 8 | 116236 | 116243 | CNS |
| 115 | p1 | (A)14 | 14 | 116841 | 116854 | CNS |
| 116 | p4 | (TAAG)3 | 12 | 117311 | 117322 | CNS |
| 117 | p3 | (ATA)4 | 12 | 118851 | 118862 | CNS |
| 118 | p5 | (AATAA)3 | 15 | 118981 | 118995 | *ndhD* |
| 119 | p1 | (A)8 | 8 | 119333 | 119340 | *ndhD* |
| 120 | p1 | (A)8 | 8 | 120125 | 120132 | *ndhD* |
| 121 | p1 | (T)8 | 8 | 120926 | 120933 | *psaC* |
| 123 | p1 | (A)9 | 9 | 121906 | 121914 | *ndhG* |
| 124 | p1 | (A)8 | 8 | 122298 | 122305 | *ndhG* |
| 125 | p1 | (T)13 | 13 | 122482 | 122494 | CNS |
| 126 | p1 | (A)8 | 8 | 124578 | 124585 | *ndhA*-CDS2 |
| 127 | p2 | (TA)8 | 16 | 126506 | 126521 | *ndhH*; *rps15* |
| 128 | p1 | (T)13 | 13 | 128094 | 128106 | *ycf1* |
| 129 | p1 | (T)9 | 9 | 128210 | 128218 | *ycf1* |
| 130 | p4 | (CATT)3 | 12 | 129078 | 129089 | *ycf1* |
| 131 | p1 | (T)10 | 10 | 129663 | 129672 | *ycf1* |
| 132 | p1 | (T)16 | 16 | 129821 | 129836 | *ycf1* |
| 134 | p1 | (T)9 | 9 | 130523 | 130531 | *ycf1* |
| 135 | p1 | (A)8 | 8 | 130636 | 130643 | *ycf1* |
| 137 | p3 | (AGT)4 | 12 | 131267 | 131278 | *ycf1* |
| 138 | p1 | (A)8 | 8 | 131743 | 131750 | *ycf1* |
| 139 | p1 | (A)8 | 8 | 133826 | 133833 | CNS |
| 140 | p1 | (T)9 | 9 | 134216 | 134224 | CNS |
| 141 | p1 | (G)9 | 9 | 141725 | 141733 | CNS |
| 142 | p1 | (T)8 | 8 | 141943 | 141950 | CNS |
| 145 | p1 | (T)9 | 9 | 152621 | 152629 | *ycf2*-D2 |
| 146 | p1 | (C)12 | 12 | 157102 | 157113 | CNS |
| 149 | p4 | (TTAG)3 | 12 | 161115 | 161126 | *rpl14*-D2; *rpl16*-D2-CDS2 |
| 150 | p1 | (A)9 | 9 | 161684 | 161692 | *rps8*-D2 |

**SSR simple sequence repeats, CDS coding sequences, CNS non-coding sequences**
