# Supplementary material for: Comparative analysis of the complete chloroplast genome sequences of six species of Pulsatilla Miller, Ranunculaceae
Source: Chin Med. 2019 Nov 28;14:53. doi: 10.1186/s13020-019-0274-5 (PMC6883693; doi:10.1186/s13020-019-0274-5)
Supplement: Supplementary file 12 — Additional file 12: Table S7. SSRs distribution of the P. turczaninovii cp genome. [file 13020_2019_274_MOESM12_ESM.docx]

**Table S7 SSRs distribution of the *P. turczaninovii* cp genome**

| **SSR nr.** | **SSR Type** | **SSR** | **Size** | **Star** | **End** | **Location** |
| --- | --- | --- | --- | --- | --- | --- |
| 3 | p2 | (TA)6 | 12 | 1594 | 1605 | *rps16*-CDS1 |
| 4 | p1 | (T)10 | 10 | 1872 | 1881 | CNS |
| 5 | p1 | (A)15 | 15 | 2256 | 2270 | CNS |
| 6 | p1 | (T)9 | 9 | 3088 | 3096 | CNS |
| 7 | p1 | (A)8 | 8 | 3201 | 3208 | CNS |
| 8 | p1 | (A)9 | 9 | 3756 | 3764 | CNS |
| 9 | p1 | (T)9 | 9 | 4012 | 4020 | CNS |
| 10 | p1 | (T)10 | 10 | 4581 | 4590 | *matK* |
| 11 | p1 | (A)9 | 9 | 5602 | 5610 | *matK* |
| 12 | p1 | (T)8 | 8 | 5926 | 5933 | *matK* |
| 13 | p1 | (T)9 | 9 | 6244 | 6252 | CNS |
| 17 | p2 | (AT)5 | 10 | 9603 | 9612 | CNS |
| 18 | p1 | (A)9 | 9 | 11418 | 11426 | *ycf3*-CDS2 |
| 19 | p1 | (A)8 | 8 | 12441 | 12448 | CNS |
| 20 | p4 | (ATTA)3 | 12 | 18177 | 18188 | CNS |
| 23 | p2 | (AT)6 | 12 | 19223 | 19234 | CNS |
| 24 | p1 | (C)10 | 10 | 20462 | 20471 | *psbC* |
| 25 | p1 | (A)8 | 8 | 22179 | 22186 | CNS |
| 26 | p5 | (ATTAA)3 | 15 | 22439 | 22453 | CNS |
| 27 | p4 | (ATCT)3 | 12 | 22674 | 22685 | CNS |
| 28 | p1 | (A)8 | 8 | 22931 | 22938 | CNS |
| 29 | p1 | (A)11 | 11 | 23364 | 23374 | CNS |
| 30 | p1 | (T)9 | 9 | 23622 | 23630 | CNS |
| 31 | p1 | (A)9 | 9 | 23820 | 23828 | CNS |
| 32 | p3 | (ATA)4 | 12 | 23954 | 23965 | CNS |
| 34 | p2 | (AT)5 | 10 | 25500 | 25509 | CNS |
| 35 | p1 | (A)9 | 9 | 25866 | 25874 | CNS |
| 36 | p1 | (A)10 | 10 | 25980 | 25989 | CNS |
| 37 | p1 | (A)8 | 8 | 28370 | 28377 | CNS |
| 38 | p1 | (A)10 | 10 | 30087 | 30096 | *rpoB* |
| 39 | p1 | (A)8 | 8 | 33757 | 33764 | CNS |
| 40 | p1 | (T)8 | 8 | 33978 | 33985 | *rpoC1*-CDS2 |
| 41 | p1 | (A)9 | 9 | 35145 | 35153 | *rpoC1*-CDS2 |
| 42 | p2 | (AT)5 | 10 | 36416 | 36425 | *rpoC2* |
| 43 | p1 | (G)8 | 8 | 37263 | 37270 | *rpoC2* |
| 44 | p1 | (T)8 | 8 | 37648 | 37655 | *rpoC2* |
| 45 | p1 | (A)14 | 14 | 37786 | 37799 | *rpoC2* |
| 46 | p1 | (A)9 | 9 | 37962 | 37970 | *rpoC2* |
| 47 | p1 | (A)8 | 8 | 38186 | 38193 | *rpoC2* |
| 48 | p1 | (T)8 | 8 | 38619 | 38626 | *rpoC2* |
| 49 | p1 | (A)8 | 8 | 39982 | 39989 | *rps2* |
| 52 | p1 | (T)8 | 8 | 43671 | 43678 | *atpF*-CDS1 |
| 53 | p1 | (A)9 | 9 | 44133 | 44141 | CNS |
| 55 | p1 | (T)11 | 11 | 46601 | 46611 | *atpA* |
| 56 | p1 | (A)8 | 8 | 47348 | 47355 | CNS |
| 57 | p1 | (T)9 | 9 | 47484 | 47492 | CNS |
| 58 | p1 | (A)8 | 8 | 48228 | 48235 | CNS |
| 59 | p1 | (A)8 | 8 | 49102 | 49109 | CNS |
| 61 | p1 | (T)9 | 9 | 51347 | 51355 | *ndhJ* |
| 62 | p1 | (A)11 | 11 | 52898 | 52908 | CNS |
| 64 | p1 | (T)13 | 13 | 54512 | 54524 | CNS |
| 65 | p1 | (T)12 | 12 | 54712 | 54723 | CNS |
| 66 | p1 | (T)8 | 8 | 54832 | 54839 | *atpE* |
| 68 | p1 | (A)17 | 17 | 57212 | 57228 | CNS |
| 69 | p1 | (T)8 | 8 | 59338 | 59345 | CNS |
| 70 | p1 | (T)8 | 8 | 60157 | 60164 | *accD* |
| 72 | p1 | (T)12 | 12 | 62059 | 62070 | *psaI* |
| 73 | p1 | (A)8 | 8 | 62440 | 62447 | *ycf4* |
| 74 | p1 | (A)8 | 8 | 63514 | 63521 | CNS |
| 75 | p1 | (T)9 | 9 | 64459 | 64467 | *cemA* |
| 76 | p1 | (A)8 | 8 | 65026 | 65033 | *petA* |
| 79 | p1 | (A)8 | 8 | 67805 | 67812 | CNS |
| 81 | p1 | (A)10 | 10 | 69412 | 69421 | *psaJ* |
| 82 | p1 | (T)8 | 8 | 69553 | 69560 | *psaJ* |
| 83 | p1 | (A)8 | 8 | 69980 | 69987 | *rpl33* |
| 87 | p1 | (T)11 | 11 | 72263 | 72273 | *rps12*-D2-CDS1; *clpP*-CDS1 |
| 89 | p1 | (A)10 | 10 | 77534 | 77543 | CNS |
| 90 | p1 | (T)19 | 19 | 79599 | 79617 | CNS |
| 91 | p1 | (A)8 | 8 | 80431 | 80438 | *rpoA* |
| 92 | p1 | (T)10 | 10 | 80660 | 80669 | *rpoA* |
| 93 | p1 | (A)8 | 8 | 81367 | 81374 | *rpoA* |
| 95 | p1 | (T)11 | 11 | 82935 | 82945 | *rps8* |
| 96 | p4 | (CTAA)3 | 12 | 83503 | 83514 | *rpl16*-CDS1; *rpl14* |
| 99 | p1 | (G)9 | 9 | 87515 | 87523 | CNS |
| 100 | p1 | (A)9 | 9 | 91996 | 92004 | *ycf2* |
| 101 | p1 | (T)8 | 8 | 101232 | 101239 | CNS |
| 103 | p1 | (A)8 | 8 | 102626 | 102633 | CNS |
| 104 | p1 | (C)9 | 9 | 102843 | 102851 | CNS |
| 105 | p1 | (A)12 | 12 | 110037 | 110048 | CNS |
| 106 | p1 | (A)9 | 9 | 110352 | 110360 | CNS |
| 107 | p1 | (T)8 | 8 | 110747 | 110754 | CNS |
| 108 | p1 | (T)8 | 8 | 112830 | 112837 | CNS |
| 109 | p3 | (TAC)4 | 12 | 113301 | 113312 | CNS |
| 111 | p1 | (A)8 | 8 | 115742 | 115749 | *ndhF* |
| 112 | p1 | (A)8 | 8 | 116283 | 116290 | CNS |
| 114 | p1 | (A)8 | 8 | 116843 | 116850 | CNS |
| 115 | p4 | (TAAG)3 | 12 | 117627 | 117638 | CNS |
| 117 | p1 | (A)8 | 8 | 119721 | 119728 | *ndhD* |
| 118 | p1 | (A)8 | 8 | 120513 | 120520 | *ndhD* |
| 119 | p1 | (T)8 | 8 | 121314 | 121321 | *psaC* |
| 121 | p1 | (A)9 | 9 | 122302 | 122310 | *ndhG* |
| 123 | p1 | (T)17 | 17 | 122875 | 122891 | CNS |
| 124 | p1 | (A)8 | 8 | 124952 | 124959 | *ndhA*-CDS2 |
| 125 | p2 | (TA)6 | 12 | 126891 | 126902 | *ndhH*; *rps15* |
| 126 | p1 | (T)13 | 13 | 128475 | 128487 | *ycf1* |
| 127 | p1 | (T)9 | 9 | 128591 | 128599 | *ycf1* |
| 128 | p4 | (CATT)3 | 12 | 129477 | 129488 | *ycf1* |
| 129 | p1 | (T)11 | 11 | 129823 | 129833 | *ycf1* |
| 130 | p1 | (T)10 | 10 | 130056 | 130065 | *ycf1* |
| 131 | p1 | (T)15 | 15 | 130214 | 130228 | *ycf1* |
| 135 | p3 | (AGT)4 | 12 | 131660 | 131671 | *ycf1* |
| 136 | p1 | (A)8 | 8 | 132136 | 132143 | *ycf1* |
| 137 | p1 | (A)8 | 8 | 134219 | 134226 | CNS |
| 138 | p1 | (T)9 | 9 | 134613 | 134621 | CNS |
| 139 | p1 | (T)12 | 12 | 134925 | 134936 | CNS |
| 140 | p1 | (G)9 | 9 | 142122 | 142130 | CNS |
| 141 | p1 | (T)8 | 8 | 142340 | 142347 | CNS |
| 143 | p1 | (A)8 | 8 | 143734 | 143741 | CNS |
| 144 | p1 | (T)9 | 9 | 152969 | 152977 | *ycf2*-D2 |
| 145 | p1 | (C)9 | 9 | 157450 | 157458 | CNS |
| 148 | p4 | (TTAG)3 | 12 | 161459 | 161470 | *rpl16*-D2-CDS2; *rpl14*-D2 |
| 149 | p1 | (A)11 | 11 | 162028 | 162038 | *rps8*-D2 |

**SSR simple sequence repeats, CDS coding sequences, CNS non-coding sequences**
