# Supplementary material for: Comparative analysis of the complete chloroplast genome sequences of six species of Pulsatilla Miller, Ranunculaceae
Source: Chin Med. 2019 Nov 28;14:53. doi: 10.1186/s13020-019-0274-5 (PMC6883693; doi:10.1186/s13020-019-0274-5)
Supplement: Supplementary file 13 — Additional file 13: Table S8. SSRs distribution of the P. cernua cp genome. [file 13020_2019_274_MOESM13_ESM.docx]

**Table S8 SSRs distribution of the *P. cernua* cp genome**

| **SSR nr.** | **SSR Type** | **SSR** | **Size** | **Star** | **End** | **Location** |
| --- | --- | --- | --- | --- | --- | --- |
| 3 | p1 | (T)9 | 9 | 1909 | 1917 | CNS |
| 4 | p1 | (A)13 | 13 | 2292 | 2304 | CNS |
| 7 | p4 | (AGAT)3 | 12 | 4343 | 4354 | *matK* |
| 8 | p1 | (T)10 | 10 | 4565 | 4574 | *matK* |
| 9 | p1 | (T)9 | 9 | 4919 | 4927 | *matK* |
| 10 | p1 | (A)9 | 9 | 5586 | 5594 | *matK* |
| 11 | p1 | (T)9 | 9 | 6197 | 6205 | CNS |
| 15 | p2 | (AT)5 | 10 | 9634 | 9643 | CNS |
| 16 | p1 | (A)8 | 8 | 11433 | 11440 | *ycf3*-CDS2 |
| 17 | p1 | (A)8 | 8 | 12463 | 12470 | CNS |
| 18 | p4 | (ATTA)3 | 12 | 18183 | 18194 | CNS |
| 21 | p2 | (AT)7 | 14 | 19254 | 19267 | CNS |
| 22 | p1 | (C)10 | 10 | 20495 | 20504 | *psbC* |
| 23 | p1 | (A)8 | 8 | 22212 | 22219 | CNS |
| 24 | p4 | (ATCT)3 | 12 | 22632 | 22643 | CNS |
| 25 | p1 | (A)9 | 9 | 22889 | 22897 | CNS |
| 26 | p1 | (A)9 | 9 | 23303 | 23311 | CNS |
| 27 | p1 | (T)10 | 10 | 23559 | 23568 | CNS |
| 28 | p3 | (ATA)4 | 12 | 23903 | 23914 | CNS |
| 30 | p2 | (AT)5 | 10 | 25467 | 25476 | CNS |
| 32 | p1 | (A)8 | 8 | 28324 | 28331 | CNS |
| 33 | p1 | (A)10 | 10 | 30035 | 30044 | *rpoB* |
| 34 | p1 | (T)8 | 8 | 33328 | 33335 | CNS |
| 35 | p1 | (T)8 | 8 | 33921 | 33928 | *rpoC1*-CDS2 |
| 36 | p1 | (A)9 | 9 | 35088 | 35096 | *rpoC1*-CDS2 |
| 37 | p2 | (AT)5 | 10 | 36359 | 36368 | *rpoC2* |
| 38 | p1 | (G)8 | 8 | 37206 | 37213 | *rpoC2* |
| 39 | p1 | (T)8 | 8 | 37591 | 37598 | *rpoC2* |
| 40 | p1 | (A)14 | 14 | 37729 | 37742 | *rpoC2* |
| 41 | p1 | (A)9 | 9 | 37905 | 37913 | *rpoC2* |
| 42 | p1 | (A)8 | 8 | 38129 | 38136 | *rpoC2* |
| 43 | p1 | (T)8 | 8 | 38562 | 38569 | *rpoC2* |
| 44 | p1 | (A)8 | 8 | 39925 | 39932 | *rps2* |
| 47 | p1 | (T)8 | 8 | 43685 | 43692 | *atpF*-CDS1 |
| 48 | p1 | (A)9 | 9 | 44147 | 44155 | CNS |
| 49 | p3 | (TAT)4 | 12 | 44403 | 44414 | CNS |
| 50 | p1 | (T)9 | 9 | 46667 | 46675 | *atpA* |
| 51 | p1 | (A)10 | 10 | 47406 | 47415 | CNS |
| 52 | p1 | (T)11 | 11 | 47544 | 47554 | CNS |
| 53 | p1 | (A)11 | 11 | 47809 | 47819 | CNS |
| 54 | p1 | (A)8 | 8 | 48290 | 48297 | CNS |
| 55 | p1 | (A)10 | 10 | 49221 | 49230 | CNS |
| 57 | p1 | (T)9 | 9 | 51498 | 51506 | *ndhJ* |
| 59 | p1 | (A)14 | 14 | 53740 | 53753 | CNS |
| 61 | p1 | (T)12 | 12 | 54690 | 54701 | CNS |
| 62 | p1 | (T)11 | 11 | 54892 | 54902 | CNS |
| 63 | p1 | (T)9 | 9 | 57054 | 57062 | *atpB* |
| 64 | p1 | (A)9 | 9 | 57428 | 57436 | CNS |
| 65 | p1 | (T)8 | 8 | 59542 | 59549 | CNS |
| 66 | p1 | (T)8 | 8 | 60355 | 60362 | *accD* |
| 67 | p1 | (A)9 | 9 | 61886 | 61894 | CNS |
| 68 | p1 | (T)11 | 11 | 62288 | 62298 | *psaI* |
| 69 | p1 | (A)8 | 8 | 62673 | 62680 | *ycf4* |
| 70 | p1 | (A)9 | 9 | 63756 | 63764 | CNS |
| 71 | p1 | (T)9 | 9 | 64702 | 64710 | *cemA* |
| 72 | p1 | (A)8 | 8 | 65269 | 65276 | *petA* |
| 73 | p1 | (A)9 | 9 | 65885 | 65893 | *petA* |
| 74 | p1 | (T)10 | 10 | 66122 | 66131 | CNS |
| 75 | p1 | (A)8 | 8 | 67949 | 67956 | CNS |
| 77 | p1 | (A)9 | 9 | 69576 | 69584 | *psaJ* |
| 79 | p1 | (A)10 | 10 | 70532 | 70541 | *rps18* |
| 82 | p1 | (T)11 | 11 | 72494 | 72504 | *clpP*-CDS1; *rps12*-D2-CDS1 |
| 84 | p1 | (T)9 | 9 | 74222 | 74230 | CNS |
| 85 | p1 | (A)13 | 13 | 77780 | 77792 | CNS |
| 86 | p4 | (TTTA)3 | 12 | 77900 | 77911 | CNS |
| 87 | p1 | (A)13 | 13 | 78081 | 78093 | CNS |
| 88 | p1 | (T)15 | 15 | 79852 | 79866 | CNS |
| 90 | p1 | (T)10 | 10 | 80910 | 80919 | *rpoA* |
| 91 | p1 | (A)8 | 8 | 81617 | 81624 | *rpoA* |
| 93 | p1 | (T)9 | 9 | 83185 | 83193 | *rps8* |
| 94 | p4 | (CTAA)3 | 12 | 83751 | 83762 | *rpl16*-CDS1; *rpl14* |
| 97 | p1 | (G)8 | 8 | 87769 | 87776 | CNS |
| 98 | p1 | (A)9 | 9 | 92249 | 92257 | *ycf2* |
| 99 | p5 | (TGTAA)3 | 15 | 100558 | 100572 | CNS |
| 100 | p1 | (T)9 | 9 | 101520 | 101528 | CNS |
| 102 | p1 | (A)8 | 8 | 102941 | 102948 | CNS |
| 103 | p1 | (C)9 | 9 | 103158 | 103166 | CNS |
| 104 | p1 | (A)9 | 9 | 110667 | 110675 | CNS |
| 105 | p1 | (T)8 | 8 | 111062 | 111069 | CNS |
| 106 | p1 | (T)8 | 8 | 113139 | 113146 | CNS |
| 107 | p3 | (TAC)4 | 12 | 113610 | 113621 | CNS |
| 110 | p1 | (T)8 | 8 | 114708 | 114715 | *ndhF* |
| 113 | p4 | (TAAG)3 | 12 | 117318 | 117329 | CNS |
| 114 | p1 | (T)8 | 8 | 118216 | 118223 | *ccsA* |
| 116 | p1 | (A)8 | 8 | 119449 | 119456 | *ndhD* |
| 117 | p1 | (A)8 | 8 | 120241 | 120248 | *ndhD* |
| 118 | p1 | (T)8 | 8 | 121042 | 121049 | *psaC* |
| 119 | p4 | (ATTA)3 | 12 | 121791 | 121802 | *ndhG* |
| 120 | p1 | (A)9 | 9 | 122032 | 122040 | *ndhG* |
| 121 | p1 | (A)8 | 8 | 122424 | 122431 | *ndhG* |
| 122 | p1 | (T)22 | 22 | 122619 | 122640 | CNS |
| 124 | p1 | (T)8 | 8 | 127256 | 127263 | CNS |
| 125 | p1 | (T)13 | 13 | 128392 | 128404 | *ycf1* |
| 126 | p1 | (T)9 | 9 | 128508 | 128516 | *ycf1* |
| 127 | p4 | (CATT)3 | 12 | 129376 | 129387 | *ycf1* |
| 128 | p1 | (T)10 | 10 | 129955 | 129964 | *ycf1* |
| 129 | p1 | (T)15 | 15 | 130113 | 130127 | *ycf1* |
| 131 | p1 | (T)9 | 9 | 130812 | 130820 | *ycf1* |
| 132 | p1 | (A)8 | 8 | 130925 | 130932 | *ycf1* |
| 133 | p1 | (A)9 | 9 | 131575 | 131583 | *ycf1* |
| 134 | p3 | (AGT)4 | 12 | 131730 | 131741 | *ycf1* |
| 135 | p1 | (A)8 | 8 | 132206 | 132213 | *ycf1* |
| 136 | p1 | (A)8 | 8 | 134283 | 134290 | CNS |
| 137 | p1 | (T)9 | 9 | 134677 | 134685 | CNS |
| 138 | p1 | (G)9 | 9 | 142186 | 142194 | CNS |
| 139 | p1 | (T)8 | 8 | 142404 | 142411 | CNS |
| 141 | p1 | (A)9 | 9 | 143824 | 143832 | CNS |
| 142 | p5 | (TTACA)3 | 15 | 144780 | 144794 | CNS |
| 143 | p1 | (T)9 | 9 | 153095 | 153103 | *ycf2*-D2 |
| 144 | p1 | (C)8 | 8 | 157576 | 157583 | CNS |
| 147 | p4 | (TTAG)3 | 12 | 161590 | 161601 | *rpl14*-D2; *rpl16*-D2-CDS2 |
| 148 | p1 | (A)9 | 9 | 162159 | 162167 | *rps8*-D2 |

**SSR simple sequence repeats, CDS coding sequences, CNS non-coding sequences**
