# Supplementary material for: Comparative analysis of the complete chloroplast genome sequences of six species of Pulsatilla Miller, Ranunculaceae
Source: Chin Med. 2019 Nov 28;14:53. doi: 10.1186/s13020-019-0274-5 (PMC6883693; doi:10.1186/s13020-019-0274-5)
Supplement: Supplementary file 14 — Additional file 14: Table S9. Large repeats identified in the P. chinensis cp genome. [file 13020_2019_274_MOESM14_ESM.docx]

**Table S9 Large repeats identified in the *P. chinensis* cp genome**

| **ID** | **Length** | **Type** | **Repeat 1 start** | **Repeat 2 start** | **Mismatch (bp)** | **E-value** | **gene** | **region** |
| --- | --- | --- | --- | --- | --- | --- | --- | --- |
| R1 | 35 | F | 61 | 134992 | 1 | 6.72E-10 | *rps4*, _ | LSC, IRb |
| R2 | 35 | P | 61 | 111168 | 1 | 6.72E-10 | *rps4*, _ | LSC, IRa |
| R3 | 33 | F | 905 | 62429 | 3 | 1.51E-05 | _ | LSC |
| R4 | 47 | F | 1005 | 62511 | 3 | 1.67E-13 | _ | LSC |
| R5 | 32 | F | 1020 | 62526 | 2 | 1.83E-06 | _ | LSC |
| R6 | 34 | F | 2937 | 2950 | 1 | 2.61E-09 | _ | LSC |
| R7 | 33 | F | 2969 | 3011 | 0 | 1.02E-10 | _ | LSC |
| R8 | 33 | F | 3022 | 113485 | 2 | 4.86E-07 | _ | LSC, SSC |
| R9 | 40 | P | 9150 | 9150 | 2 | 4.38E-11 | _ | LSC |
| R10 | 30 | F | 9201 | 48748 | 0 | 6.55E-09 | *trnS-GGA*,  *trnS-GCU* | LSC |
| R11 | 39 | F | 10885 | 145123 | 0 | 2.50E-14 | _ | LSC, IRb |
| R12 | 39 | P | 10885 | 101033 | 0 | 2.50E-14 | _ | LSC, IRa |
| R13 | 52 | F | 13691 | 15915 | 3 | 2.22E-16 | *psaA*, *psaB* | LSC |
| R14 | 32 | F | 13711 | 15935 | 2 | 1.83E-06 | *psaA*, *psaB* | LSC |
| R15 | 35 | F | 23215 | 23239 | 3 | 1.13E-06 | _ | LSC |
| R16 | 32 | F | 24464 | 24479 | 0 | 4.09E-10 | _ | LSC |
| R17 | 30 | F | 24637 | 24677 | 1 | 5.89E-07 | _ | LSC |
| R18 | 30 | P | 43423 | 43423 | 2 | 2.56E-05 | _ | LSC |
| R19 | 33 | F | 52967 | 52976 | 2 | 4.86E-07 | _ | LSC |
| R20 | 33 | F | 53674 | 113641 | 3 | 1.51E-05 | _ | LSC |
| R21 | 49 | P | 76849 | 76849 | 3 | 1.19E-14 | _ | LSC |
| R22 | 31 | F | 94000 | 94036 | 1 | 1.52E-07 | *ycf2* | IRa |
| R23 | 31 | P | 94000 | 152128 | 1 | 1.52E-07 | *ycf2*, *ycf2-*D2 | \| IRa, IRb \| \| --- \| |
| R24 | 49 | F | 94000 | 94018 | 1 | 3.50E-18 | *ycf2* | IRa |
| R25 | 49 | P | 94000 | 152128 | 1 | 3.50E-18 | *ycf2*, *ycf2-*D2 | \| IRa, IRb \| \| --- \| |
| R26 | 49 | P | 94018 | 152146 | 1 | 3.50E-18 | *ycf2*, *ycf2-*D2 | \| IRa, IRb \| \| --- \| |
| R27 | 31 | P | 94036 | 152164 | 1 | 1.52E-07 | *ycf2*, *ycf2-*D2 | \| IRa, IRb \| \| --- \| |
| R28 | 33 | P | 113474 | 113483 | 3 | 1.51E-05 | _ | SSC |
| R29 | 31 | F | 113530 | 113555 | 2 | 6.85E-06 | _ | SSC |
| R30 | 37 | F | 113631 | 113653 | 1 | 4.44E-11 | _ | SSC |
| R31 | 34 | F | 113685 | 113703 | 3 | 4.13E-06 | _ | SSC |
| R32 | 32 | R | 113713 | 114070 | 2 | 1.83E-06 | _ | SSC |
| R33 | 37 | F | 113795 | 113829 | 2 | 2.40E-09 | _ | SSC |
| R34 | 32 | R | 113887 | 113890 | 1 | 3.93E-08 | _ | SSC |
| R35 | 36 | R | 113888 | 113961 | 3 | 3.08E-07 | _ | SSC |
| R36 | 33 | R | 113910 | 113910 | -2 | 4.86E-07 | _ | SSC |
| R37 | 59 | F | 113978 | 114028 | 0 | 2.27E-26 | _ | SSC |
| R38 | 33 | C | 114009 | 116614 | 3 | 1.51E-05 | _ | SSC |
| R39 | 31 | F | 114016 | 114101 | 2 | 6.85E-06 | _ | SSC |
| R40 | 38 | C | 114097 | 116611 | 3 | 2.28E-08 | _ | SSC |
| R41 | 31 | R | 114190 | 114190 | 2 | 6.85E-06 | *ndhF* | SSC |
| R42 | 33 | R | 116576 | 116584 | 3 | 1.51E-05 | _ | SSC |
| R43 | 32 | F | 120035 | 120060 | 1 | 3.93E-08 | _ | SSC |
| R44 | 49 | P | 121860 | 121860 | 1 | 3.50E-18 | _ | SSC |
| R45 | 30 | F | 125853 | 125878 | 1 | 5.89E-07 | _ | SSC |
| R46 | 32 | R | 127832 | 127832 | 2 | 1.83E-06 | _ | SSC |
| R47 | 40 | R | 127873 | 127873 | 2 | 4.38E-11 | _ | SSC |
| R48 | 31 | F | 152128 | 152164 | 1 | 1.52E-07 | *ycf2-*D2 | IRb |
| R49 | 49 | F | 152128 | 152146 | 1 | 3.50E-18 | *ycf2-*D2 | IRb |

**F forward, P palindromic, C complement, R reverse, - intergenic space**
