# Supplementary material for: Comparative analysis of the complete chloroplast genome sequences of six species of Pulsatilla Miller, Ranunculaceae
Source: Chin Med. 2019 Nov 28;14:53. doi: 10.1186/s13020-019-0274-5 (PMC6883693; doi:10.1186/s13020-019-0274-5)
Supplement: Supplementary file 16 — Additional file 16: Table S11. Large repeats identified in the P. cernua f. plumbea cp genome. [file 13020_2019_274_MOESM16_ESM.docx]

**Table S11 Large repeats identified in the *P. cernua* f. *plumbea* cp genome**

| **ID** | **Length** | **Type** | **Repeat 1 start** | **Repeat 2 start** | **Mismatch (bp)** | **E-value** | **gene** | **region** |
| --- | --- | --- | --- | --- | --- | --- | --- | --- |
| R1 | 35 | F | 61 | 133570 | 1 | 6.60E-10 | *rps4*, _ | LSC, IRb |
| R2 | 35 | P | 61 | 110801 | 1 | 6.60E-10 | *rps4*, _ | LSC, IRa |
| R3 | 33 | F | 890 | 62153 | 3 | 1.48E-05 | _ | LSC |
| R4 | 37 | F | 1000 | 62245 | 3 | 8.25E-08 | _ | LSC |
| R5 | 31 | F | 1220 | 1264 | 1 | 1.50E-07 | _ | LSC |
| R6 | 36 | F | 1245 | 1265 | -3 | 3.03E-07 | _ | LSC |
| R7 | 36 | F | 3222 | 3241 | 3 | 3.03E-07 | _ | LSC |
| R8 | 40 | P | 9172 | 9172 | 0 | 6.14E-15 | _ | LSC |
| R9 | 32 | P | 9221 | 19305 | 3 | 5.39E-05 | *trnS-GGA*, *trns-UGA* | LSC |
| R10 | 30 | F | 9223 | 48436 | 0 | 6.44E-09 | *trnS-GGA*, *trns-GCU* | LSC |
| R11 | 39 | F | 10919 | 143738 | 0 | 2.46E-14 | _ | LSC, IRb |
| R12 | 39 | P | 10919 | 100629 | 0 | 2.46E-14 | _ | LSC, IRa |
| R13 | 52 | F | 13712 | 15936 | 3 | 2.18E-16 | *psaA*, *psaB* | LSC |
| R14 | 32 | F | 13732 | 15956 | 2 | 1.80E-06 | *psaA*, *psaB* | LSC |
| R15 | 30 | F | 18206 | 46621 | 3 | 7.06E-04 | *trnG-GCC*, *trnG-UCC* | LSC |
| R16 | 30 | P | 19305 | 48436 | 3 | 7.06E-04 | *trnS-UGA*, *trns-GCU* | LSC |
| R17 | 30 | R | 24408 | 118973 | 3 | 7.06E-04 | _ | LSC, SSC |
| R18 | 32 | P | 48324 | 48324 | 2 | 1.80E-06 | _ | LSC |
| R19 | 37 | R | 49030 | 49030 | 2 | 2.36E-09 | _ | LSC |
| R20 | 30 | P | 53425 | 79340 | 3 | 7.06E-04 | _ | LSC |
| R21 | 49 | P | 76470 | 76470 | 3 | 1.17E-14 | _ | LSC |
| R22 | 31 | F | 93574 | 93610 | 1 | 1.50E-07 | *ycf2* | IRa |
| R23 | 31 | P | 93574 | 150765 | 1 | 1.50E-07 | *ycf2*, *ycf2-*D2 | IRa, IRb |
| R24 | 49 | F | 93574 | 93592 | 1 | 3.44E-18 | *ycf2* | IRa |
| R25 | 49 | P | 93574 | 150765 | 1 | 3.44E-18 | *ycf2*, *ycf2-*D2 | IRa, IRb |
| R26 | 49 | P | 93592 | 150783 | 1 | 3.44E-18 | *ycf2*, *ycf2-*D2 | IRa, IRb |
| R27 | 31 | P | 93610 | 150801 | 1 | 1.50E-07 | *ycf2*, *ycf2-*D2 | IRa, IRb |
| R28 | 30 | F | 111003 | 133373 | 2 | 2.52E-05 | _ | IRa, IRb |
| R29 | 30 | P | 111003 | 111003 | 2 | 2.52E-05 | _ | IRa |
| R30 | 38 | R | 115714 | 115714 | 2 | 6.22E-10 | _ | SSC |
| R31 | 30 | R | 116703 | 126530 | 3 | 7.06E-04 | _ | SSC |
| R32 | 31 | P | 118751 | 118784 | 3 | 1.95E-04 | _ | SSC |
| R33 | 49 | P | 120601 | 120601 | 3 | 1.17E-14 | _ | SSC |
| R34 | 30 | C | 126523 | 126524 | 3 | 7.06E-04 | _ | SSC |
| R35 | 30 | F | 130870 | 130894 | 3 | 7.06E-04 | *ycf1* | SSC |
| R36 | 30 | P | 133373 | 133373 | 2 | 2.52E-05 | _ | IRb |
| R37 | 31 | F | 150765 | 150801 | 1 | 1.50E-07 | *ycf2*-D2 | IRb |
| R38 | 49 | F | 150765 | 150783 | 1 | 3.44E-18 | *ycf2*-D2 | IRb |

**F forward, P palindromic, C complement, R reverse, - intergenic space**
