# Supplementary material for: Comparative analysis of the complete chloroplast genome sequences of six species of Pulsatilla Miller, Ranunculaceae
Source: Chin Med. 2019 Nov 28;14:53. doi: 10.1186/s13020-019-0274-5 (PMC6883693; doi:10.1186/s13020-019-0274-5)
Supplement: Supplementary file 19 — Additional file 19: Table S14. Large repeats identified in the P. cernua cp genome. [file 13020_2019_274_MOESM19_ESM.docx]

**Table S14 Large repeats identified in the *P. cernua* cp genome**

| **ID** | **Length** | **Type** | **Repeat 1 start** | **Repeat 2 start** | **Mismatch (bp)** | **E-value** | **gene** | **region** |
| --- | --- | --- | --- | --- | --- | --- | --- | --- |
| R1 | 35 | F | 61 | 133932 | 1 | 6.63E-10 | *rps4*, _ | LSC, IRb |
| R2 | 35 | P | 61 | 111007 | 1 | 6.63E-10 | *rps4*, _ | LSC, IRb |
| R3 | 33 | F | 899 | 62319 | 3 | 1.49E-05 | _ | LSC |
| R4 | 47 | F | 999 | 62401 | 3 | 1.65E-13 | _ | LSC |
| R5 | 32 | F | 1014 | 62416 | 2 | 1.80E-06 | _ | LSC |
| R6 | 30 | R | 1097 | 1098 | 1 | 5.82E-07 | _ | LSC |
| R7 | 34 | P | 1150 | 1150 | 2 | 1.28E-07 | _ | LSC |
| R8 | 30 | F | 1175 | 1195 | 3 | 7.09E-04 | _ | LSC |
| R9 | 31 | F | 1215 | 1279 | 2 | 6.76E-06 | _ | LSC |
| R10 | 31 | F | 1221 | 1260 | 3 | 1.96E-04 | _ | LSC |
| R11 | 30 | F | 3236 | 3283 | 3 | 7.09E-04 | _ | LSC |
| R12 | 40 | P | 9272 | 9272 | 2 | 4.33E-11 | _ | LSC |
| R13 | 32 | P | 9321 | 19404 | 3 | 5.41E-05 | _, *trnS-UGA* | LSC |
| R14 | 30 | F | 9323 | 48725 | 0 | 6.47E-09 | _ | LSC |
| R15 | 39 | F | 11014 | 144077 | 0 | 2.47E-14 | _ | LSC, IRb |
| R16 | 39 | P | 11014 | 100858 | 0 | 2.47E-14 | _, *rps12* | LSC, IRa |
| R17 | 52 | F | 13820 | 16044 | 3 | 2.19E-16 | *psaA*, *psaB* | LSC |
| R18 | 32 | F | 13840 | 16064 | 2 | 1.80E-06 | *psaA*, *psaB* | LSC |
| R19 | 30 | F | 18312 | 46899 | 3 | 7.09E-04 | _ | LSC |
| R20 | 30 | P | 19404 | 48725 | 3 | 7.09E-04 | *trnS-UGA*, _ | LSC |
| R21 | 33 | P | 22431 | 22431 | 3 | 1.49E-05 | _ | LSC |
| R22 | 30 | P | 24611 | 52924 | 3 | 7.09E-04 | _ | LSC |
| R23 | 31 | R | 24611 | 65851 | 3 | 1.96E-04 | _ | LSC |
| R24 | 31 | F | 24695 | 143131 | 2 | 6.76E-06 | _ | LSC, IRb |
| R25 | 31 | P | 24695 | 101812 | 2 | 6.76E-06 | _ | LSC, IRa |
| R26 | 37 | R | 44337 | 44337 | 2 | 2.37E-09 | _ | LSC |
| R27 | 33 | R | 52934 | 70269 | 3 | 1.49E-05 | _, *rpl33* | LSC |
| R28 | 31 | F | 52975 | 52999 | 2 | 6.76E-06 | _ | LSC |
| R29 | 31 | P | 57353 | 70276 | 3 | 1.96E-04 | _, *rpl33* | LSC |
| R30 | 50 | R | 65788 | 65788 | 0 | 5.88E-21 | *petA* | LSC |
| R31 | 34 | P | 65830 | 65830 | 0 | 2.53E-11 | _ | LSC |
| R32 | 49 | P | 76725 | 76725 | 3 | 1.17E-14 | _ | LSC |
| R33 | 31 | R | 79596 | 122865 | 3 | 1.96E-04 | _, *ndhI* | LSC, SSC |
| R34 | 31 | F | 93825 | 93861 | 1 | 1.50E-07 | *ycf2* | IRa |
| R35 | 31 | P | 93825 | 151082 | 1 | 1.50E-07 | *ycf2*, *ycf2*-D2 | IRa, IRb |
| R36 | 49 | F | 93825 | 93843 | 1 | 3.46E-18 | *ycf2* | IRa |
| R37 | 49 | P | 93825 | 151082 | 1 | 3.46E-18 | *ycf2*, *ycf2*-D2 | IRa, IRb |
| R38 | 49 | P | 93843 | 151100 | 1 | 3.46E-18 | *ycf2*, *ycf2*-D2 | IRa, IRb |
| R39 | 31 | P | 93861 | 151118 | 1 | 1.50E-07 | *ycf2*, *ycf2*-D2 | IRa, IRb |
| R40 | 30 | F | 111209 | 133735 | 2 | 2.53E-05 | _, *trnN-GUU* | IRa, IRb |
| R41 | 30 | P | 111209 | 111209 | 2 | 2.53E-05 | _ | IRa |
| R42 | 30 | F | 119138 | 119178 | 2 | 2.53E-05 | *ndhD* | SSC |
| R43 | 30 | R | 119197 | 119197 | 2 | 2.53E-05 | *ndhD* | SSC |
| R44 | 31 | F | 119212 | 119244 | 3 | 1.96E-04 | *ndhD* | SSC |
| R45 | 49 | P | 120958 | 120958 | 1 | 3.46E-18 | *psaC* | SSC |
| R46 | 31 | F | 131551 | 131575 | 3 | 1.96E-04 | *ycf1* | IRb |
| R47 | 30 | P | 133735 | 133735 | 2 | 2.53E-05 | *trnN-GUU* | IRb |
| R48 | 31 | F | 151082 | 151118 | 1 | 1.50E-07 | *ycf2*-D2 | IRb |
| R49 | 49 | F | 151082 | 151100 | 1 | 3.46E-18 | *ycf2*-D2 | IRb |

**F forward, P palindromic, C complement, R reverse, - intergenic space**
