# Supplementary material for: Comparative analysis of the complete chloroplast genome sequences of six species of Pulsatilla Miller, Ranunculaceae
Source: Chin Med. 2019 Nov 28;14:53. doi: 10.1186/s13020-019-0274-5 (PMC6883693; doi:10.1186/s13020-019-0274-5)
Supplement: Supplementary file 20 — Additional file 20: Table S15. Pi values of the coding and no-coding regions in the six Pulsatilla cp genomes. [file 13020_2019_274_MOESM20_ESM.docx]

**Table S15 Pi values of the coding and no-coding regions in the six *Pulsatilla* cp genomes**

| **Coding region** | | | **No-coding region** | | |
| --- | --- | --- | --- | --- | --- |
| **Gene** | **Length** | **Pi** | **Gene** | **Length** | **Pi** |
| *rps4* | 630 | 0.0005291 | *start-rps4* | 2 | 0 |
| *rps16*-CDS1 | 40 | 0 | *rps4-rps16*-CDS1 | 591 | 0.0157924 |
| *rps16*-CDS2 | 194 | 0 | *rps16*-CDS1-*rps16*-CDS2 | 883 | 0.0049830 |
| *matK* | 1527 | 0.0024012 | *rps16*-CDS2-*matK* | 1605 | 0.0161163 |
| *psbA* | 1062 | 0.0010672 | *matK-psbA* | 569 | 0.0058582 |
| *psbK* | 186 | 0 | *psbA-psbK* | 929 | 0.0097596 |
| *psbI* | 111 | 0 | *psbK-psbI* | 428 | 0.0049844 |
| *ycf3*-CDS1 | 124 | 0 | *psbI-ycf3*-CDS1 | 1048 | 0.0056616 |
| *ycf3*-CDS2 | 230 | 0.0014493 | *ycf3*-CDS1-*ycf3*-CDS2 | 727 | 0.0023842 |
| *ycf3*-CDS3 | 153 | 0 | *ycf3*-CDS2-*ycf3*-CDS3 | 760 | 0.0051754 |
| *psaA* | 2253 | 0.0012428 | *ycf3*-CDS3-*psaA* | 697 | 0.0008608 |
| *psaB* | 2205 | 0.0005442 | *psaA-psaB* | 27 | 0 |
| *rps14* | 303 | 0 | *psaB-rps14* | 131 | 0.0040712 |
| *psbZ* | 189 | 0 | *rps14-psbZ* | 894 | 0.0046234 |
| *psbC* | 1422 | 0.0019691 | *psbZ-psbC* | 640 | 0.0028125 |
| *psbD* | 1062 | 0.0020088 | *psbD-psbM* | 4025 | 0.0136646 |
| *psbM* | 105 | 0 | *psbM-petN* | 1198 | 0.0036728 |
| *petN* | 90 | 0 | *petN-rpoB* | 1673 | 0.0038255 |
| *rpoB* | 3213 | 0.0009960 | *rpoB-rpoC1*-CDS1 | 28 | 0 |
| *rpoC1*-CDS1 | 430 | 0.0007752 | *rpoC1*-CDS1-*rpoC1*-CDS2 | 755 | 0.0018543 |
| *rpoC1*-CDS2 | 1609 | 0.0005801 | *rpoC1*-CDS2-*rpoC2* | 176 | 0.0018939 |
| *rpoC2* | 4152 | 0.0017341 | *rpoC2-rps2* | 196 | 0.0054422 |
| *rps2* | 708 | 0.0007533 | *rps2-atpI* | 222 | 0.0042042 |
| *atpI* | 744 | 0.0007168 | *atpI-atpH* | 1133 | 0.0057664 |
| *atpH* | 246 | 0 | *atpH-atpF*-CDS1 | 462 | 0.0079365 |
| *atpF*-CDS1 | 145 | 0 | *atpF*-CDS1-*atpF*-CDS2 | 780 | 0.0116239 |
| *atpF*-CDS2 | 410 | 0.0013008 | *atpF*-CDS2-*atpA* | 68 | 0.0078431 |
| *atpA* | 1524 | 0.0007874 | *atpA-ndhJ* | 4290 | 0.0056099 |
| *ndhJ* | 477 | 0.0006988 | *ndhJ-ndhK* | 140 | 0 |
| *ndhK* | 684 | 0.0046784 | *ndhK-ndhC* | 60 | 0 |
| *ndhC* | 363 | 0.0009183 | *ndhC-atpE* | 2185 | 0.0067735 |
| *atpE* | 402 | 0.0013267 | *atpB-rbcL* | 813 | 0.0063141 |
| *atpB* | 1503 | 0.0005766 | *rbcL-accD* | 737 | 0.0036183 |
| *rbcL* | 1428 | 0.0010271 | *accD-psaI* | 657 | 0.0054795 |
| *accD* | 1437 | 0.0020413 | *psaI-ycf4* | 473 | 0.0031008 |
| *psaI* | 111 | 0 | *ycf4-cemA* | 637 | 0.0046049 |
| *ycf4* | 555 | 0 | *cemA-petA* | 206 | 0 |
| *cemA* | 690 | 0.0025121 | *petA-psbJ* | 419 | 0.0198886 |
| *petA* | 969 | 0.0026832 | *psbJ-psbL* | 121 | 0.0027548 |
| *psbJ* | 123 | 0 | *psbL-psbF* | 24 | 0.0222222 |
| *psbL* | 117 | 0 | *psbF-psbE* | 11 | 0 |
| *psbF* | 120 | 0 | *psbE-petL* | 1234 | 0.0055646 |
| *psbE* | 252 | 0 | *petL-petG* | 180 | 0.0140741 |
| *petL* | 95 | 0.0035088 | *petG-psaJ* | 829 | 0.0082831 |
| *petG* | 112 | 0 | *psaJ-rpl33* | 430 | 0.0068217 |
| *psaJ* | 135 | 0.0024691 | *rpl33-rps18* | 184 | 0.0365942 |
| *rpl33* | 201 | 0.0016584 | *rps18-rpl20* | 240 | 0.0130556 |
| *rps18* | 306 | 0 | *rpl20-rps12*-CDS1 | 783 | 0.0007663 |
| *rpl20* | 354 | 0.0009416 | *rps12*-CDS1-*clpP*-CDS3 | 149 | 0.0035794 |
| *rps12*-CDS1 | 114 | 0 | *clpP*-CDS3-*clpP*-CDS2 | 711 | 0.0067511 |
| *clpP*-CDS3 | 246 | 0.0035230 | *clpP*-CDS2-*clpP*-CDS1 | 780 | 0.0019658 |
| *clpP*-CDS2 | 289 | 0.0018454 | *clpP*-CDS1-*psbB* | 404 | 0.0013201 |
| *clpP*-CDS1 | 71 | 0 | *psbB-psbT* | 193 | 0.0055268 |
| *psbB* | 1527 | 0.0012661 | *psbT-psbN* | 104 | 0.0096154 |
| *psbT* | 108 | 0 | *psbN-psbH* | 101 | 0.0052805 |
| *psbN* | 132 | 0 | *psbH-petB*-CDS1 | 121 | 0.0027548 |
| *psbH* | 222 | 0 | *petB*-CDS1-*petB*-CDS2 | 791 | 0.0092710 |
| *petB*-CDS1 | 6 | 0 | *petB*-CDS2-*petD*-CDS1 | 204 | 0.0026144 |
| *petB*-CDS2 | 642 | 0.0005192 | *petD*-CDS1-*petD*-CDS2 | 746 | 0.0063450 |
| *petD*-CDS1 | 8 | 0 | *petD*-CDS2-*rpoA* | 184 | 0.0083333 |
| *petD*-CDS2 | 496 | 0.0020161 | *rpoA-rps11* | 80 | 0.0091667 |
| *rpoA* | 996 | 0.0024096 | *rps11-rpl36* | 115 | 0.0052174 |
| *rps11* | 417 | 0.0043165 | *rpl36-rps8* | 308 | 0.0021645 |
| *rpl36* | 114 | 0.0140351 | *rps8-rpl14* | 219 | 0.0024353 |
| *rps8* | 399 | 0 | *rpl14-rpl16-*CDS2 | 144 | 0 |
| *rpl14* | 369 | 0 | *rpl16*-CDS2-*rpl16*-CDS1 | 977 | 0.0006141 |
| *rpl16*-CDS2 | 399 | 0.0013367 | *rpl16*-CDS1-*rps3* | 175 | 0 |
| *rpl16*-CDS1 | 9 | 0 | *rpl22-rps19* | 61 | 0 |
| *rps3* | 657 | 0 | *rps19-rpl2*-CDS2 | 62 | 0 |
| *rpl22* | 546 | 0 | *rpl2*-CDS2-*rpl2*-CDS1 | 663 | 0.0005028 |
| *rps19* | 279 | 0 | *rpl2*-CDS1-*rpl23* | 20 | 0 |
| *rpl2*-CDS2 | 434 | 0 | *rpl23-ycf2* | 309 | 0 |
| *rpl2*-CDS1 | 391 | 0 | *ycf2-ndhB*-CDS2 | 1265 | 0 |
| *rpl23* | 282 | 0 | *ndhB*-CDS2-*ndhB-*CDS1 | 708 | 0 |
| *ycf2* | 6825 | 0.0003907 | *ndhB*-CDS1-*rps7* | 325 | 0 |
| *ndhB*-CDS2 | 756 | 0.0007055 | *rps7-rps12*-CDS3 | 55 | 0 |
| *ndhB*-CDS1 | 777 | 0.0004290 | *rps12*-CDS3-*rps12*-CDS2 | 542 | 0 |
| *rps7* | 468 | 0 | *rps12*-CDS2-*ndhF* | 12599 | 0.0024182 |
| *rps12*-CDS3 | 26 | 0 | *ndhF-ccsA* | 1203 | 0.0114159 |
| *rps12*-CDS2 | 232 | 0 | *ccsA-ndhD* | 343 | 0.0575316 |
| *ndhF* | 2197 | 0.0025793 | *ndhD-psaC* | 132 | 0.0065657 |
| *ccsA* | 966 | 0.0045549 | *psaC-ndhE* | 246 | 0.0043360 |
| *ndhD* | 1503 | 0.0034154 | *ndhE-ndhG* | 227 | 0.0114537 |
| *psaC* | 246 | 0.0021680 | *ndhG-ndhI* | 411 | 0.0095702 |
| *ndhE* | 306 | 0.0019608 | *ndhI-ndhA*-CDS2 | 75 | 0.0044444 |
| *ndhG* | 534 | 0.0037453 | *ndhA*-CDS2-*ndhA*-CDS1 | 849 | 0.0016490 |
| *ndhI* | 542 | 0.0012300 | *ndhA*-CDS1-*ndhH* | 3 | 0 |
| *ndhA*-CDS2 | 539 | 0.0016079 | *ndhH-rps15* | 141 | 0.0316785 |
| *ndhA*-CDS1 | 553 | 0.0028933 | *rps15-ycf1* | 410 | 0.0013008 |
| *ndhH* | 1182 | 0.0011844 | *ycf1-rps12*-D2-CDS2 | 10799 | 0.0005000 |
| *rps15* | 273 | 0 | *rps12*-D2-CDS2-*rps12*-D2-CDS3 | 542 | 0 |
| *ycf1* | 5625 | 0.0058311 | *rps12*-D2-CDS3-*rps7*-D2 | 55 | 0 |
| *rps12*-D2-CDS2 | 232 | 0 | *rps7*-D2-*ndhB-*D2-CDS1 | 325 | 0 |
| *rps12*-D2-CDS3 | 26 | 0 | *ndhB*-D2-CDS1-*ndhB*-D2-CDS2 | 708 | 0 |
| *rps7*-D2 | 468 | 0 | *ndhB*-D2-CDS2-*ycf2*-D2 | 1265 | 0 |
| *ndhB*-D2-CDS1 | 777 | 0.0004290 | *ycf2*-D2-*rpl23*-D2 | 309 | 0 |
| *ndhB*-D2-CDS2 | 756 | 0.0007055 | *rpl23*-D2-*rpl2*-D2-CDS1 | 20 | 0 |
| *ycf2*-D2 | 6825 | 0.0003907 | *rpl2*-D2-CDS1-*rpl2-*D2-CDS2 | 663 | 0.0005028 |
| *rpl23*-D2 | 282 | 0 | *rpl2*-D2-CDS2-*rps19*-D2 | 62 | 0 |
| *rpl2*-D2-CDS1 | 391 | 0 | *rps19*-D2-*rpl22*-D2 | 61 | 0 |
| *rpl2*-D2-CDS2 | 434 | 0 | *rps3*-D2-*rpl16*-D2-CDS1 | 175 | 0 |
| *rps19*-D2 | 279 | 0 | *rpl16*-D2-CDS1-*rpl16*-D2-CDS2 | 977 | 0.0006141 |
| *rpl22*-D2 | 546 | 0 | *rpl16*-D2-CDS2-*rpl14*-D2 | 144 | 0 |
| *rps3*-D2 | 657 | 0 | *rpl14-*D2-*rps8*-D2 | 219 | 0.0024353 |
| *rpl16*-D2-CDS1 | 9 | 0 | *rps8*-D2 | 303 | 0.0022002 |
| *rpl16*-D2-CDS2 | 399 | 0.0013367 | *-* | - | - |
| *rpl14*-D2 | 369 | 0 | *-* | - | - |
| *rps8*-D2 | 399 | 0 | *-* | - | - |
